# Molecular characterization of the calcium release channel deficiency syndrome

**DOI:** 10.1172/jci.insight.135952

**Published:** 2020-08-06

**Authors:** David J. Tester, CS John Kim, Samantha K. Hamrick, Dan Ye, Bailey J. O’Hare, Hannah M. Bombei, Kristi K. Fitzgerald, Carla M. Haglund-Turnquist, Dianne L. Atkins, Luis A. Ochoa Nunez, Ian Law, Joel Temple, Michael J. Ackerman

**Affiliations:** 1 Windland Smith Rice Sudden Death Genomics Laboratory, Department of Molecular Pharmacology and Experimental Therapeutics; Division of Heart Rhythm Services, Department of Cardiovascular Medicine; and Division of Pediatric Cardiology, Department of Pediatric and Adolescent Medicine, Mayo Clinic, Rochester, Minnesota, USA.; 2 Division of Cardiology, University of Iowa Stead Family Children’s Hospital, Iowa City, Iowa, USA.; 3 Nemours Cardiac Center, Nemours/Alfred I. duPont Hospital for Children, Wilmington, Delaware, USA.

**Keywords:** Cardiology, Genetics, Cardiovascular disease, Genetic variation, Ion channels

## Abstract

We identified a potentially novel homozygous duplication involving the promoter region and exons 1–4 of the gene encoding type 2 cardiac ryanodine receptor (*RYR2*) that is responsible for highly penetrant, exertion-related sudden deaths/cardiac arrests in the Amish community without an overt phenotype to suggest *RYR2-*mediated catecholaminergic polymorphic ventricular tachycardia (CPVT). Homozygous RYR2 duplication (RYR2-DUP) induced pluripotent stem cell cardiomyocytes (iPSC-CMs) were generated from 2 unrelated patients. There was no difference in baseline Ca^2+^ handling measurements between WT-iPSC-CM and RYR2-DUP-iPSC-CM lines. However, compared with WT-iPSC-CMs, both patient lines demonstrated a dramatic reduction in caffeine-stimulated and isoproterenol-stimulated (ISO-stimulated) Ca^2+^ transient amplitude, suggesting RyR2 loss of function. There was a greater than 50% reduction in *RYR2* transcript/RyR2 protein expression in both patient iPSC-CMs compared with WT. Delayed afterdepolarization was observed in the RYR2-DUP-iPSC-CMs but not in the WT-iPSC-CMs. Compared with WT-iPSC-CMs, there was significantly elevated arrhythmic activity in the RYR2-DUP-iPSC-CMs in response to ISO. Nadolol, propranolol, and flecainide reduced erratic activity by 8.5-fold, 6.8-fold, and 2.4-fold, respectively, from ISO challenge. Unlike the gain-of-function mechanism observed in *RYR2-*mediated CPVT, the homozygous multiexon duplication precipitated a dramatic reduction in *RYR2* transcription and RyR2 protein translation, a loss of function in calcium handling, and a calcium-induced calcium release apparatus that is insensitive to catecholamines and caffeine.

## Abstract 2

Molecular and functional characterization of the calcium release channel deficiency syndrome in patient-specific induced pluripotent stem cell-cardiomyocytes.

## Graphical abstract

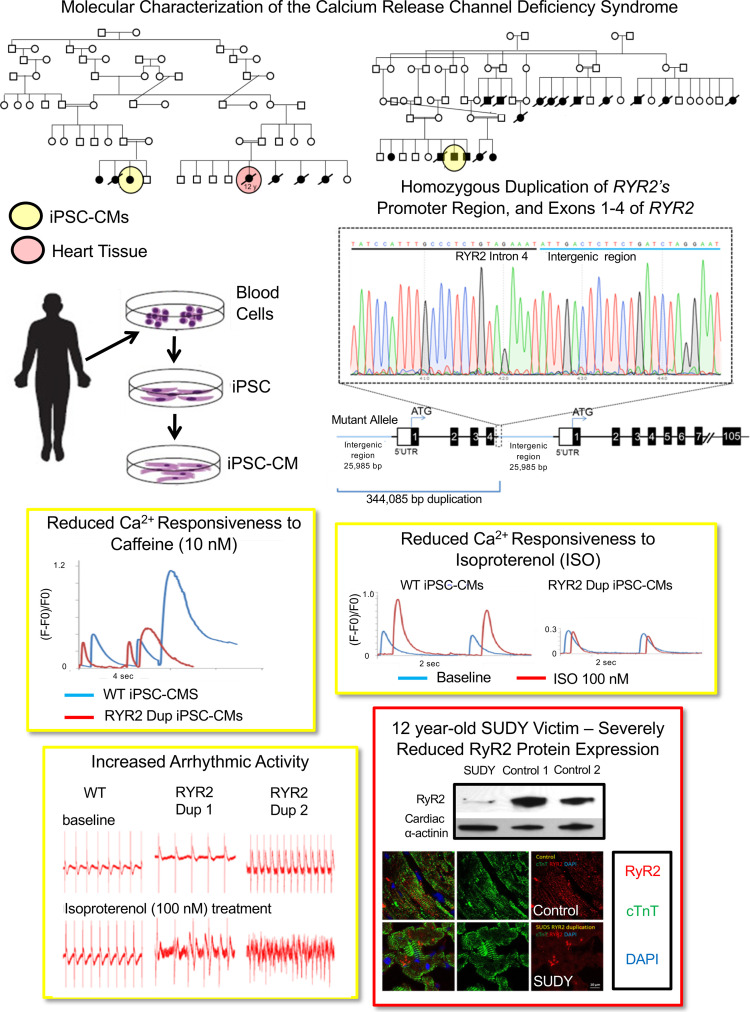

## Introduction

The *RYR2* gene encodes for the protein known as the type 2 cardiac ryanodine receptor (RyR2)/calcium release channel (CRC), containing 4967 amino acids and 105 exons, that is a critical component of the normal calcium-induced calcium release (CICR) mechanism of excitation-contraction coupling in cardiomyocytes (CMs) (1). In ventricular CMs, the depolarization of the sarcolemma by influx of sodium (Na^+^) through the cardiac sodium channel (Nav1.5) leads to opening of voltage-dependent L-type calcium channels (LTCCs) during the plateau phase of the cardiac action potential. The entry of a small amount of Ca^2+^ into the cell triggers the massive release of Ca^2+^ from the sarcoplasmic reticulum (SR), the principal intracellular Ca^2+^ storage organelle of the cell, through RyR2s/CRCs. This cytosolic Ca^2+^ bathes the contractile filaments, binds to and activates protein troponin C (the Ca^2+^-sensing protein of the contractile apparatus), and induces myofilament contraction. Upon relaxation, the majority of the cytosolic Ca^2+^ is rapidly sequestered back into the SR by the SR Ca^2+^ ATPase (SERCA2a) or out into the extracellular space by the Na^+^/Ca^2+^ exchanger (1).

RyR2 gain-of-function (GOF) pathogenic variants account for 60% of autosomal dominant type 1 catecholaminergic polymorphic ventricular tachycardia (CPVT1), a potentially lethal, heritable arrhythmia syndrome that classically manifests as exercise-induced syncope, sudden cardiac arrest (SCA), or sudden unexplained death in the young (SUDY) in the setting of a structurally normal heart (2–4).

Previously, using a combination of exome sequencing and copy number variant (CNV) familial triangulation analysis, we identified the same large, homozygous, biallelic tandem duplication of 344,085 base pairs (bp) involving approximately 26,000 bp of intergenic sequence, *RYR2*’s 5′ UTR/promoter region, and exons 1–4 of *RYR2* as a potentially novel genetic basis for recessively inherited, exercise-associated SCA/SUDY in 2, seemingly unrelated, large multigenerational Amish pedigrees (5).

## Results

**Figure 1:**
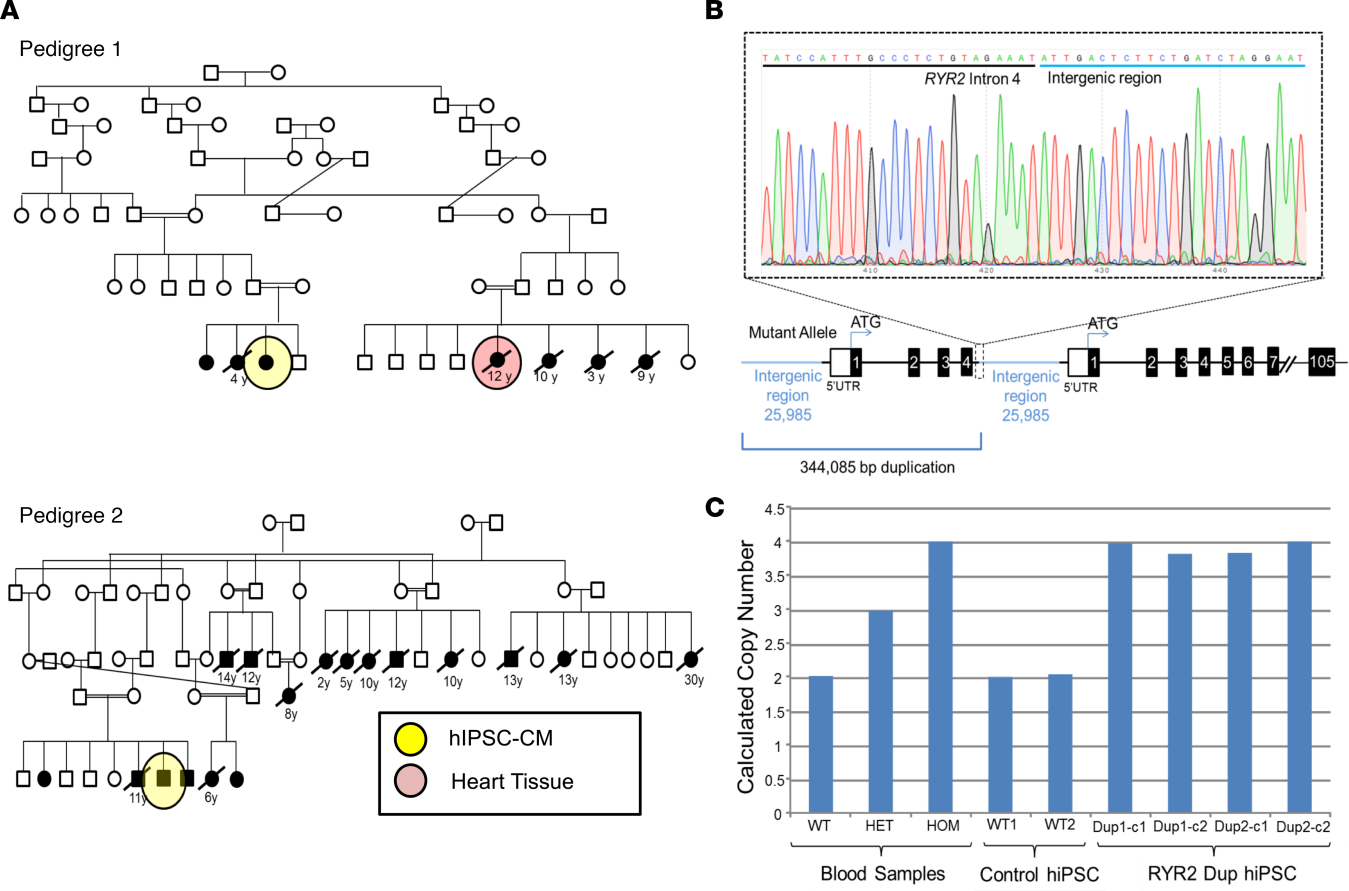
Amish pedigrees with recessively inherited exertion-associated SUDY. Shown are 2 unrelated Amish pedigrees (pedigree 1 and pedigree 2) (**A**) with autopsy-negative sudden unexplained deaths or cardiac arrests. Open symbols (circles, women and girls; squares, men and boys) represent unaffected individuals. Black symbols represent affected family members. The age (in years) at sudden death is provided below the symbol representing sex. The yellow circles represent those family members whose iPSC-CMs were available for study. The red circle indicates the sudden death victim whose heart tissue was available for study. (**B**) A representative Sanger sequencing chromatogram from one of the RYR2-duplicated (RYR2 Dup) iPSC clones hosting the homozygous duplication and a graphical representation of the biallelic tandem 344,085 base pair (bp) duplication involving approximately 26,000 bp of intergenic sequence, *RYR2*’s 5′ UTR/promoter region, and exons 1–4 of *RYR2*. (**C**) Bar graph illustrating the calculated copy number of *RYR2* alleles in genomic DNA derived from patients known to be negative (WT, 2 copies), heterozygous (3 copies), or homozygous (4 copies) for the RYR2 duplication as well as confirmation of genotype in each control and mutant iPSC clone.

Here, we characterize functionally patient-specific induced pluripotent stem cell cardiomyocytes (iPSC-CMs) derived from 2 of the affected children, one from each pedigree (Figure 1A). Clinical evaluations, including echocardiography, serial 12-lead ECGs, and exercise stress testing, were essentially normal in both patient 1 and patient 2. The clinical descriptions of both patients and the other affected relatives were reported previously (5). Patient 1 was a girl who experienced syncope after exiting a swimming pool at age 15 (patient 1, pedigree 1, Figure 1A). Her most recent normal exercise stress test was 3 weeks before her syncopal event. She has displayed subsequently some monomorphic ectopy on exercise. Two of her siblings have experienced exertion-related SCA, one of which died at age 5 years. Patient 2 was a 10-year-old boy who survived an SCA while playing baseball (patient 2, pedigree 2, Figure 1A). He has subsequently experienced 3 appropriate ventricular tachycardia/ventricular fibrillation–terminating implantable cardioverter defibrillator shocks. Expansion efforts of pedigree 2 revealed a 6-generation pedigree with over 250 family members identified. A total of 15 family members have experienced either an exertion-related youthful (≤30 years of age) sudden cardiac death (*n* = 13, 5 male, 8 female; average age at death 11.7± 6.8 years) or survival of an SCA (*n* = 2, 1 male and 1 female). Both sets of parents for patient 1 and patient 2 were heterozygous for the *RYR2* duplication, were asymptomatic, and had normal cardiologic tests.

### Generation of RYR2 duplication patient-specific iPSCs and iPSC-CMs from both patients.

**Figure 2:**
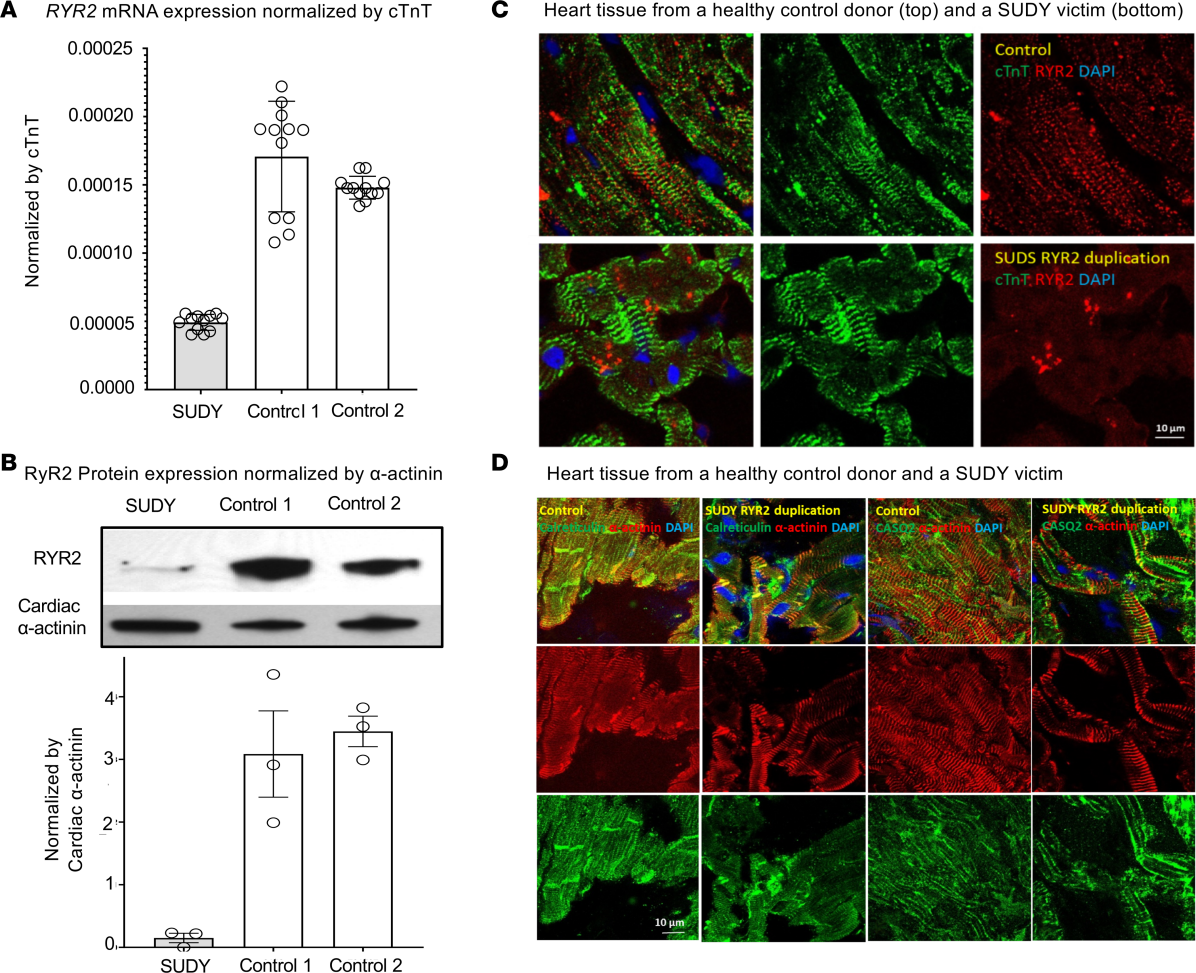
Reduced RyR2 mRNA and protein in a family member with sudden death. Shown is (**A**) real-time quantitative PCR (RT-qPCR) of *RYR2* mRNA transcript normalized by cardiac troponin (cTnT) for 2 control donor heart tissue samples (control 1, a 42-year-old woman; and control 2, a 39-year-old man) and a heart tissue sample for a family member, a 12-year-old girl with sudden unexplained death in the young (SUDY) who was homozygous for the RYR2 duplication iPSC-CMs. Two independent RT-qPCR experiments with 6 technical replicates each (*N* = 12) and (**B**) representative Western blots with RyR2 and cardiac α-actinin antibodies from the family member with SUDY and 2 unrelated control donor heart tissue samples (3 independent Western blots per sample). Shown are immunofluorescence (IF) images of a section of heart tissue collected from a relative with SUDY (pedigree 1, Figure 1) who died suddenly during exertion and a healthy 42-year-old female heart donor. (**C**) The individual IF images of RyR2 (red) and the cardiac marker cTnT (green) and the merged image along with DAPI staining for both the control and SUDY victim. (**D**) The individual IF images of the SR marker proteins calreticulin (green) and calsequestrin-2 (CASQ2, green) and the cardiac marker α-actinin (red) and the merged image along with DAPI staining for both the control and SUDY victim. Data are presented as mean ± SEM.

PBMCs were collected and used for iPSC generation for the 2 aforementioned unrelated patients who survived an exertion-associated SCA and are homozygous for the *RYR2* duplication as well as for 2 unrelated healthy controls who are negative for the *RYR2* duplication. Two clones from each patient line (RYR2 Dup 1, clones 1 and 2; and RYR2 Dup 2, clones 1 and 2) and 1 clone from each of the 2 controls (WT1, 38-year-old White woman; and WT2, 17-year-old White female young adult) were used in this study. All 4 mutant iPSC clones tested demonstrated normal karyotypes (Supplemental Figure 1). Sanger sequencing confirmed the presence of the *RYR2* duplication in each of the mutant clones from both patient lines. Figure 1B shows a representative sequence chromatogram that depicts the precise nucleotide breakpoint of the duplication. A commercially available TaqMan assay demonstrated that each iPSC mutant clone from the 2 unrelated affected individuals and the unaffected controls was either homozygous or negative for the *RYR2* duplication, respectively (Figure 1C). Undifferentiated *RYR2* duplication iPSCs were characterized by pluripotent markers with immunofluorescence (IF) imaging using a confocal microscope (Figure 2). A conventional cardiac differentiation method was used. Cardiospecific IF imaging was performed (Supplemental Figure 2). The overall morphology and shape of the iPSC-CMs was indistinguishable between the mutant lines and the unrelated control lines.

### RYR2 transcript and RyR2 protein levels are attenuated markedly in the patients’ RYR2 duplication iPSC-CMs.

The level of *RYR2* transcript was reduced significantly to just 31.5% ± 4.5% (*P* < 0.004) of control levels in the RYR2 duplication iPSC-CMs from both affected individuals (Supplemental Figure 3A). Western blot imaging revealed significantly reduced levels of RyR2 protein in the RYR2 duplication iPSC-CMs from both affected individuals to just 18% ± 3% of control levels normalized by cardiac α-actinin (Supplemental Figure 3, B and C).

### Reduced RyR2 protein level in a postmortem heart tissue sample from a family member who had sudden death.

A section of heart tissue was obtained from a 12-year-old female relative (pedigree 1) who died suddenly during exertion (Figure 1A). Compared with RNA isolated from heart tissue from 2 donor control hearts (control 1, 42-year-old woman; and control 2, 39-year-old man), the level of *RYR2* transcript observed in the relative with SUDY was reduced to just 30.9% ± 3.6% of control levels (Figure 2A). Western blot analysis revealed substantially reduced levels of RyR2 protein in the family member with SUDY compared with the control donor hearts to just 4.6% ± 4.0% of control levels (Figure 2B). Using quantitative IF, a dramatic reduction of RyR2 protein to 34.2% ± 3.7% of control levels was visualized in the victim’s heart section when compared with normal heart sections of a 42-year-old female heart donor (Figure 2C), while the SR marker proteins calreticulin and calsequestrin-2 were unaffected (Figure 2D).

### Baseline Fluo-4–measured Ca^2+^ handling measurements were unaffected in the RYR2 duplication iPSC-CMs.

**Figure 3:**
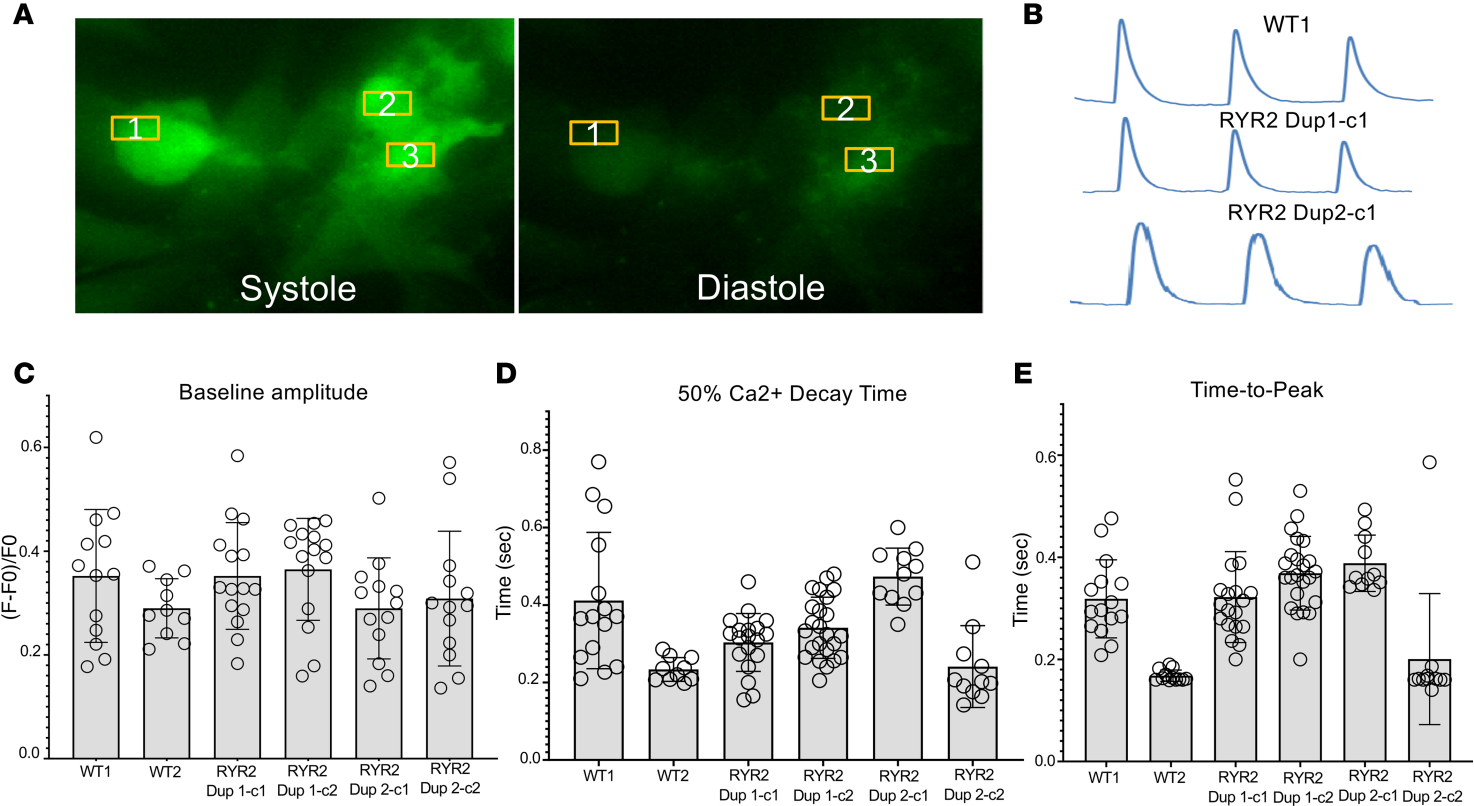
Fluo-4–measured calcium transient comparison. Shown are (**A**) screenshots of representative iPSC-CMs imaged and corresponding regions of interest used for calcium handling assessment and (**B**) representative raw tracings from unrelated WT (WT1) control iPSC-CMs and both patient human iPSC-CM lines (RYR2 Dup 1 clone 1 and clone 2 and RYR2 Dup 2 clone 1). Also shown are summary data bar graphs of (**C**) Fluo-4–measured calcium transient amplitude, normalized by (F – F0)/F0, (**D**) calcium transient decay 50% (τ), and (**E**) calcium transient time-to-peak values for (WT1 and WT2) control iPSC-CMs and both patient iPSC-CM lines (RYR2 Dup 1 clone 1 and clone 2 and RYR2 Dup 2 clone 1 and clone 2). Data are presented as mean ± standard deviation. *n* = 7 to 24 per group (Table 1).

Differentiated iPSC-CMs from both RYR2 duplication patient lines (RYR2 Dup 1, clones 1 and 2; and RYR2 Dup 2, clones 1 and 2) and WT control iPSC-CMs (WT1 and WT2) were tested with Fluo-4 based Ca^2+^ imaging. The [Ca^2+^]_i_ transient amplitude normalized by (F – F0)/F0, the 50% [Ca^2+^]_i_ transient decay time, and the [Ca^2+^]_i_ transient upstroke time to peak in the iPSC-CMs from both patient RYR2 duplication lines were indistinguishable from WT control iPSC-CMs under baseline conditions (Table 1 and Figure 3, A–E).

### Reduced Ca^2+^ responsiveness to β-adrenergic stimulation by isoproterenol in RYR2 duplication iPSC-CMs.

**Figure 4:**
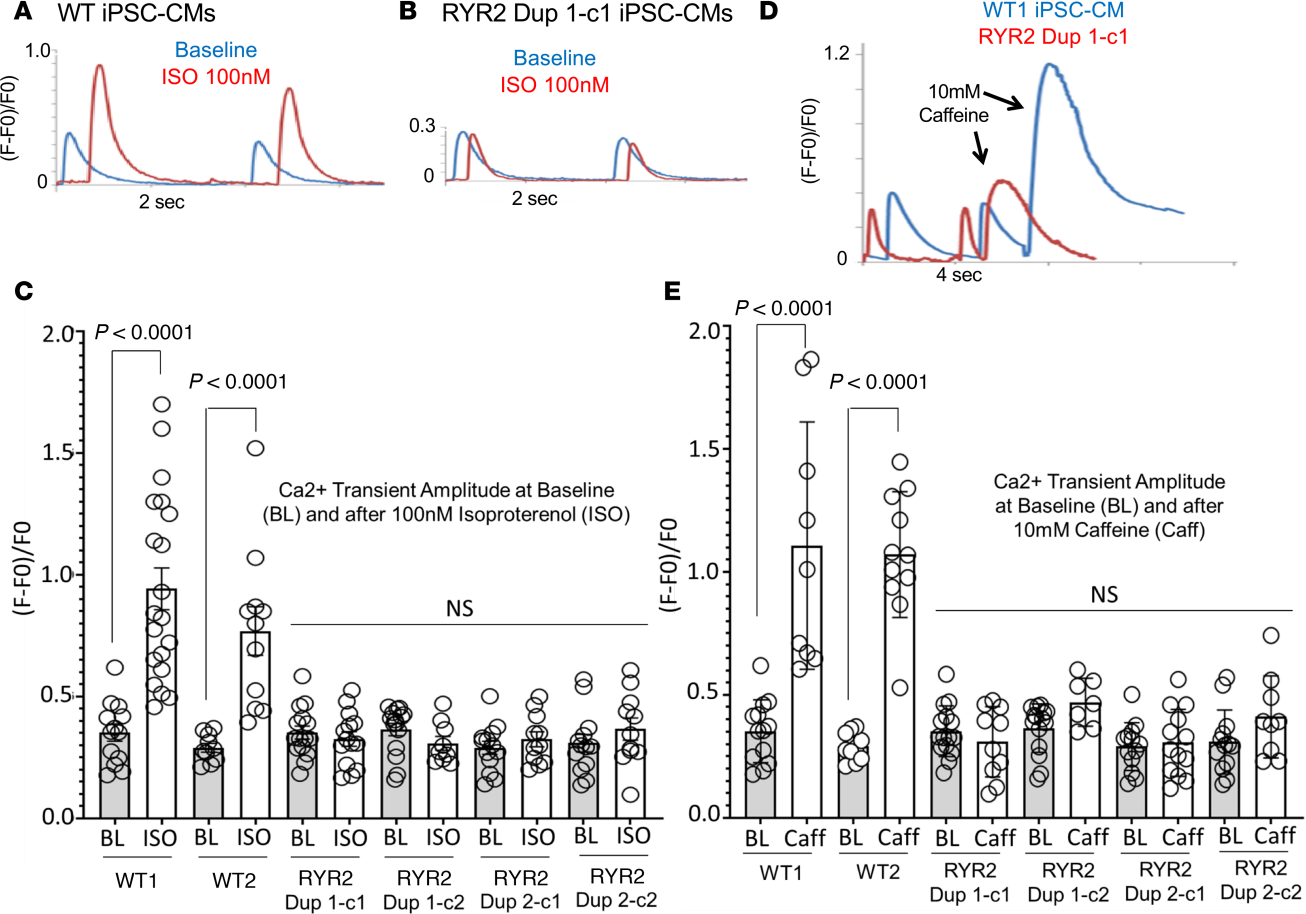
Reduced Ca^2+^ response in RYR2 duplication iPSC-CMs to ISO and to caffeine compared with control iPSC-CMs. Representative Fluo-4–measured calcium transient before (blue trace) and after 100 nM ISO (red trace) are shown for (**A**) WT (WT1) control iPSC-CMs and (**B**) the homozygous RYR2 duplication iPSC-CMs for patient 1. (**C**) The average calcium transient amplitude summary data at baseline and after 100 nM ISO treatment for the WT1 and WT2 controls and both patient iPSC-CMs (2 clones each). Representative Fluo-4–measured calcium transients before and after 10 mM caffeine are shown for (**D**) WT1 control iPSC-CMs (blue trace) and the homozygous RYR2 duplication iPSC-CMs for patient 1 (red trace). (**E**) The average calcium transient amplitude summary data at baseline (BL) and after 10 mM caffeine (Caff) treatment for the WT1 and WT2 controls and both patient iPSC-CMs (2 clones each). Data are presented as mean ± SEM (Table 1). A 2-tailed Student’s *t* test was performed to determine statistical significance between 2 groups. *P* < 0.05 was considered to be significant.

Given the multiple exertion-triggered adverse cardiac events in both patients and several family members who died suddenly during exercise, the nonspecific β-adrenergic agonist isoproterenol (ISO) was used in Fluo-4 Ca^2+^ recording to discern any intracellular calcium handling abnormalities during β-adrenergic stimulation. As expected, 100 nM ISO elicited shortening of the [Ca^2+^]_i_ transient upstroke peak time and [Ca^2+^]_i_ transient decay time in WT control iPSC-CMs (Table 1 and Figure 4A) and both RYR2 Dup 1 and RYR2 Dup 2 patient iPSC-CMs (Table 1 and Figure 4B). While the expected significant ISO-mediated [Ca^2+^]_i_ transient amplitude change as normalized by (F – F0)/F0 was observed in the WT control iPSC-CMs (WT1: 0.35 ± 0.18, *n* = 13 to 0.94 ± 0.38, *n* = 20; WT2: 0.29 ± 0.06, *n* = 10 to 0.77 ± 0.33, *n* = 11), the patient RYR2 duplication iPSC-CMs (RYR2 Dup 1 clone 1: 0.35 ± 0.10, *n* = 15 to 0.33 ± 0.11, *n* = 14; RYR2 Dup 1 clone 2: 0.36 ± 0.098, *n* = 15 to 0.31 ± 0.086, *n* = 9; RYR2 Dup 2 clone 1: 0.29 ± 0.098, *n* = 13 to 0.324 ± 0.102, *n* = 11; RYR2 Dup 2 clone 2: 0.308 ± 0.129, *n* = 13 to 0.366 ± 0.149, *n* = 11) did not elicit any normal ISO-mediated amplitude change (i.e., no response to ISO) (Table 1 and Figure 4, A–C).

### Reduced Ca^2+^ responsiveness to caffeine in RYR2 duplication iPSC-CMs.

Next, we analyzed the Ca^2+^ storage/release capabilities of the mutant and control iPSC-CMs using caffeine-mediated (10 mM) RyR2 activation. Both control iPSC-CMs (WT1 and WT2) showed the expected abrupt and dramatic increase in intracellular Ca^2+^ amplitude after caffeine treatment (Table 1 and Figure 4, D and E). However, both RYR2 duplication patient iPSC-CMs (RYR2 Dup 1, clones 1 and 2; and RYR2 Dup 2, clones 1 and 2) displayed a dramatic reduction in the responsiveness to caffeine, with no significant change in [Ca^2+^]_i_ transient amplitude after caffeine treatment (Table 1 and Figure 4, D and E), suggesting significantly reduced response to caffeine/RyR2-mediated SR Ca^2+^ release.

### Single-cell action potential recordings revealed the presence of delayed afterdepolarizations in 2 independent patients’ iPSC-CMs but not in control iPSC-CMs.

**Figure 5:**
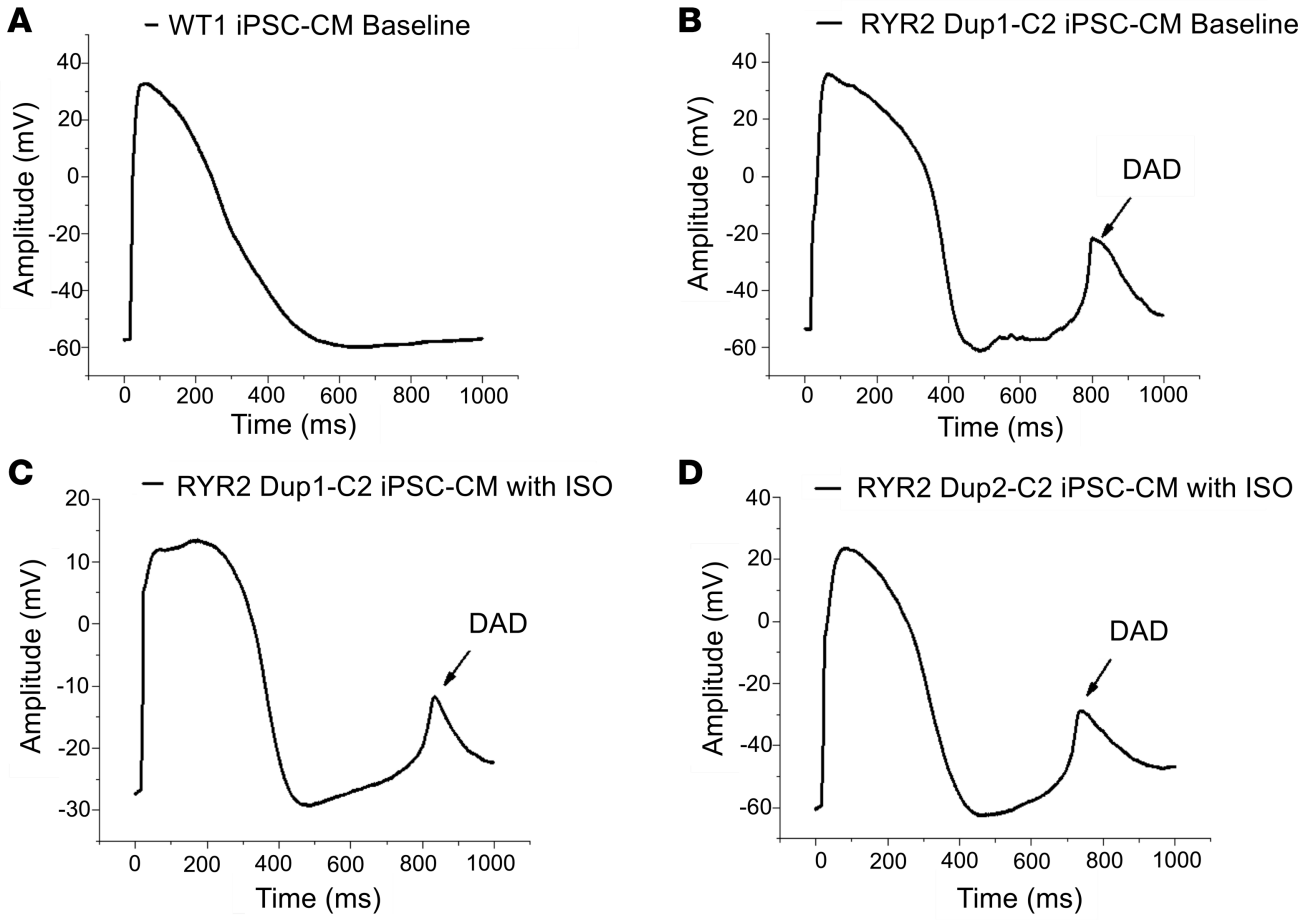
Action potential recordings by patch-clamp showing DAD events in RYR2 duplication iPSC-CMs. Representative action potential traces from (**A**) WT (WT1) control iPSC-CMs (*n* = 10) and (**B**) RYR2 Dup 1-c2 mutant iPSC-CMs under baseline conditions (*n* = 10) and (**C**) RYR2 Dup 1-c2 mutant (*n* = 8) and (**D**) RYR2 Dup 2-c2 mutant (*n* = 5) iPSC-CMs following ISO (100 nM) treatment. DAD events are indicated by the arrow.

Delayed afterdepolarizations (DADs) were not observed in any control iPSC-CMs (Figure 5A). However, the RYR2 Dup 1 clone 2 (Dup 1-c2) mutant iPSC-CMs revealed 1 episode of DAD in 1 cell without ISO (Figure 5B) and 1 episode of DAD in another cell with 100 nM ISO perfusion (Figure 5C). One episode of DAD was also shown in 1 of the RYR2 Dup 2-c2 iPSC-CMs with 100 nM ISO perfusion (Figure 5D). There were no significant differences among control, RYR2 Dup 1-c2, and RYR2 Dup 2-c2 iPSC-CMs in terms of maximum diastolic potential, amplitude, or action potential duration (APD) at 50% of repolarization. However, RYR2 Dup 2-c2 iPSC-CMs revealed a significantly prolonged APD90 compared with control iPSC-CMs (612 ± 69 ms vs. 421 ± 46 ms, *P* < 0.05, Supplemental Table 1).

### Field potential measurement reveals arrhythmic activity in both patients’ iPSC-CMs but not in control iPSC-CMs.

**Figure 6:**
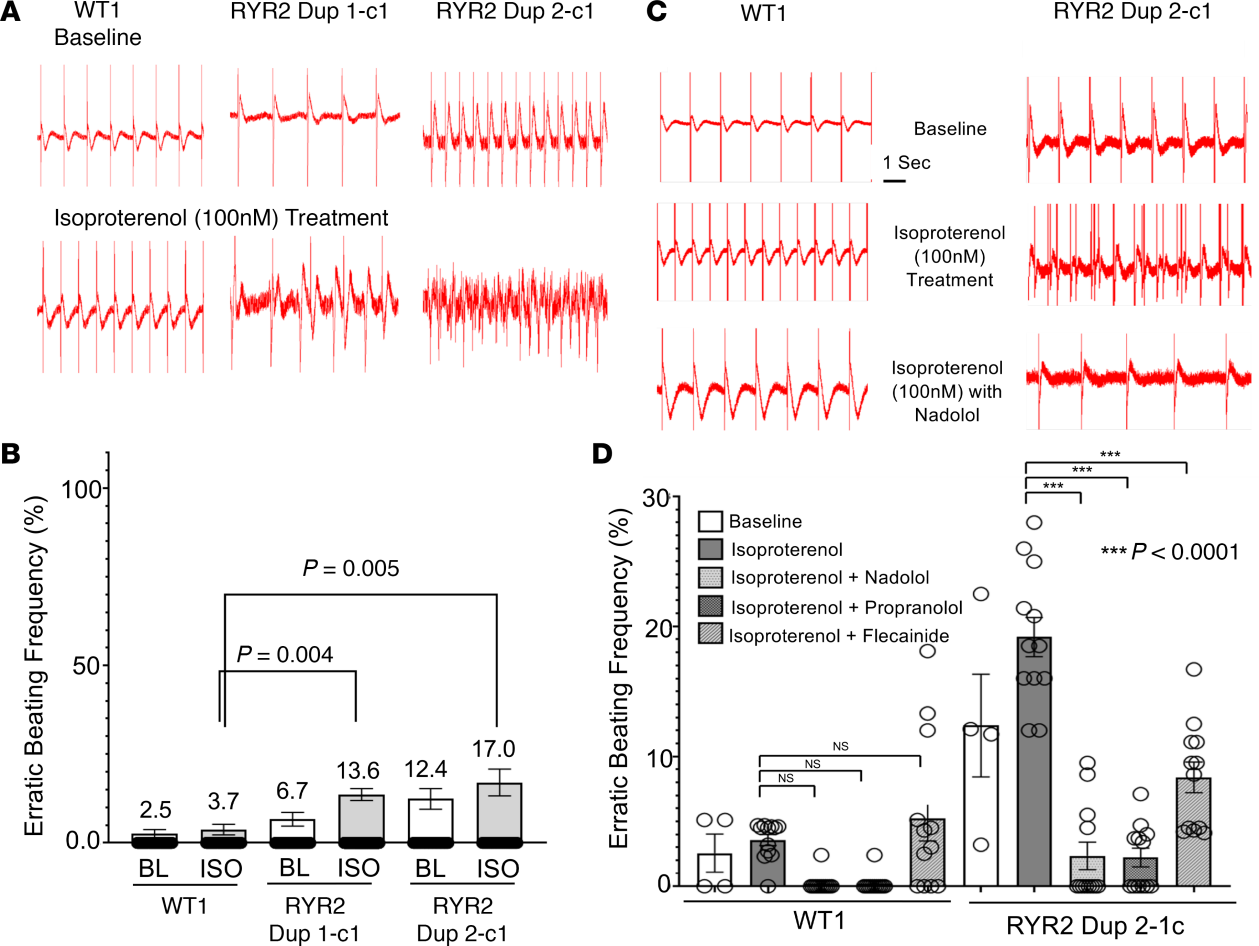
Field potential recording–based arrhythmic activity measurement. (**A**) Representative field potential (FP) recordings from WT (WT1) and the homozygous RYR2 duplication iPSC-CMs for both patients (RYR2 Dup 1 and RYR2 Dup 2) at baseline (top) and following ISO (100 nM) treatment (bottom). (**B**) A bar graph summary showing the erratic beating frequency (i.e., arrhythmic events) present at baseline and following ISO treatment in WT1 iPSC-CMs compared with RYR2 duplication iPSC-CMs for both patients. WT1-iPSC-CM baseline (*n* = 158, SEM = 1.25), WT-iPSC-CM ISO (*n* = 160, SEM = 1.5), RYR2 Dup 1-c1-iPSC-CM baseline (*n* = 165, SEM = 1.9), RYR2 Dup 1-c1-iPSC-CM ISO (*n* = 419, SEM = 1.7), RYR2 Dup 2-c1-iPSC-CM baseline (*n* = 129, SEM = 2.9), RYR2 Dup 2-c1-iPSC-CM ISO (*n* = 100, SEM = 3.8). (**C**) Representative FP recordings from WT1 control and RYR2 duplication iPSC-CMs from patient 2 (RYR2 Dup 2 clone 1) at baseline, following ISO (100 nM) treatment alone, and following ISO with nadolol (10 μM). (**D**) A bar graph summary of the erratic beating frequency (i.e., arrhythmic events) present in WT1 iPSC-CMs compared with RYR2 duplication iPSC-CMs from patient 2 (RYR2 Dup 2 clone 1) at baseline, following ISO (100 nM) and in response to pharmacotherapies (nadolol at 10 μM, propranolol at 1 μM, and flecainide at 6 μM). Data are shown as number of experiments, where each experiment includes data acquired from 250–500 electrode recordings each. WT1-iPSC-CM baseline (*n* = 4, SEM = 1.5), ISO (*n* = 12, SEM = 0.4), ISO + nadolol (*n* = 12, SEM = 0.20), ISO + propranolol (*n* = 12, SEM = 0.20), and ISO + flecainide (*n* = 12, SEM = 1.7). RYR2 Dup 2-c1-iPSC-CM baseline (*n* = 4, SEM = 3.9), ISO (*n* = 12, SEM = 1.5), ISO + nadolol (*n* = 12, SEM = 1.1), ISO + propranolol (*n* = 12, SEM = 0.72), and ISO + flecainide (*n* = 12, SEM = 1.2). Data are presented as mean ± SEM. The symbol *** represents *P* < 0.0001. A 1-way ANOVA with Tukey’s test was performed to determine statistical significance between multiple groups. *P* < 0.05 was considered significant.

The collective behavior of iPSC-CM clusters was tested using the xCELLigence RTCA CardioECR instrument (referred to as CardioECR). One week after plating iPSC-CMs in a CardioECR multielectrode plate, a recording of field potential with a minimum of 0.1 mV amplitude could be obtained (Figure 6A). There was no statistically significant difference in the percentage of arrhythmic events between the WT control and RYR2 duplication cases (3% in WT vs. 6% in RYR2 Dup 1-c1 and 9% in RYR2 Dup 2-c1) iPSC-CMs during baseline recordings (Figure 6B). However, a significantly elevated arrhythmic activity compared with WT control was observed in the RYR2 duplication patient iPSC-CMs, with 13.6% of RYR2 Dup 1-c1 (*P* = 0.004) and 17% of RYR2 Dup 2-c1 (*P* = 0.005) wells showing erratic activity in response to 100 nM ISO challenge (Figure 6B). We also noticed a similar response, albeit a weaker alternation of erratic beating activity, from *RYR2* siRNA 1– and siRNA 2–treated WT1 iPSC-CMs compared with scramble RNA–treated WT1 iPSC-CMs (Supplemental Figure 4, A and B). Both *RYR2* siRNA 1 and siRNA 2 reduced *RYR2* transcript level in WT1 iPSC-CMs to less than 50% compared with scramble RNA–treated WT1 iPSC-CMs, indicating the reduction of RYR2 causing electrical anomaly in beating CMs (Supplemental Figure 4C).

### Propranolol, nadolol, and flecainide attenuate the ISO-triggered arrhythmic activity.

Given the association of exertion-induced cardiac arrest/sudden cardiac death in these patients and their deceased relatives, increased excitation of adrenergic activity was speculated to cause potentially lethal ventricular arrhythmia. Therefore, the beta blockers nadolol and propranolol, which are preferentially used clinically for CPVT1, were tested for their efficacy in the treatment of the arrhythmic events observed in the RYR2 Dup 2-c1 iPSC-CMs because they presented with the most prevalent erratic beating frequency. Both nadolol and propranolol reduced erratic activity by 8.5-fold and 6.8-fold, respectively, from ISO challenge. In fact, propranolol and nadolol treatment during ISO challenge in RYR2 duplication iPSC-CMs resulted in fewer arrhythmic events than present in WT control even during baseline recording. The efficacy of flecainide, a class Ic Na^+^ channel blocker also used for the treatment of CPVT1, was tested in our current setting. Flecainide appeared to be slightly less potent than the beta blockers but had a significant rescue effect in arrhythmia, with a 2.4-fold reduction in erratic activity during ISO challenge (Figure 6, C and D).

## Discussion

GOF pathogenic variants in *RYR2* cause autosomal dominant CPVT1, a potentially lethal, heritable arrhythmia syndrome that classically manifests as exercise-induced syncope or SCA in the setting of a structurally normal heart (2–4). Classically, CPVT1 is associated with a normal resting ECG and is suspected clinically based on the patient’s personal and/or family history and documentation of significant and progressive ventricular ectopy during either treadmill or catecholamine stress testing, which may culminate in its trademark arrhythmia of exercise-associated bidirectional ventricular tachycardia (VT) or polymorphic VT (6). These CPVT1-causative variants often result in leaky CRCs, leading to excessive calcium release, particularly during sympathetic stimulation. This can precipitate diastolic calcium overload, DADs, and potentially lethal ventricular arrhythmias (7). While greater than 90% of *RYR2* mutations discovered to date are missense mutations, heterozygous CNVs involving the in-frame deletion of *RYR2* exon 3 (35 amino acids, Asn57_Gly91) that also leads to an overall GOF of RyR2 have been reported to cause a wide spectrum of clinical phenotypes, including autosomal dominant CPVT1, sinoatrial node dysfunction, bradycardia, atrial fibrillation, atrioventricular block, dilated cardiomyopathy, left ventricular noncompaction, and SCA (3, 8–11).

Recently, we described 2 large multigenerational Amish pedigrees with a highly penetrant and lethal, autosomal recessively inherited *RYR2* pathogenic substrate (5). Among the 2 pedigrees hosting the identical large homozygous tandem duplication involving the promoter region and exons 1–4 of *RYR2*, 23 affected individuals have been identified so far, with 22/23 (96%) being symptomatic and 18/23 (78%) dying suddenly (5). Unlike classical CPVT1, individuals homozygous for the *RYR2* duplication have typically displayed only intermittently prolonged QT intervals or prominent U-waves and mild ventricular ectopy or had a completely normal exercise stress test/epinephrine challenge or 24-hour Holter monitor (5). While phenotypically the family resembled exertion-related CPVT1, we speculated that the underlying pathology was different mechanistically and may represent a novel loss of function of RyR2, which we referred to as calcium release channel deficiency syndrome.

In 2002, Priori and colleague presented the clinical and molecular characterization of patients with CPVT (6). While 87% of their 30 probands displayed either bidirectional VT (*n* = 14, 47%) or polymorphic VT (*n* = 12, 40%), 4/30 (13%) probands were classified as having “catecholaminergic idiopathic ventricular fibrillation (cIVF),” defined as ventricular fibrillation elicited by physical or emotional stress in the absence of an identifiable precipitating factor and in the absence of VT documented at Holter and/or exercise stress testing (6). Thus, some patients diagnosed with “CPVT” may not show inducible arrhythmias on exercise stress testing despite experiencing an unexplained cardiac arrest during physical or emotional stress.

Interestingly, 1 of the 4 patients classified as having cIVF (i.e., negative Holter and/or exercise stress test), a 7-year-old girl presenting with syncope, had the p.A4860G-RyR2 variant that was demonstrated most recently to confer a loss of function (LOF) to the RyR2/CRC and lead to the formation of early afterdepolarizations (EADs) (12). In fact, 3 heterozygous RyR2 LOF missense mutations, p.I4855M, p.A4860G, and p.S4938F, have now been demonstrated recently to cause autosomal dominant left ventricular noncompaction with atypical CPVT, cIVF, or “short-coupled torsades de pointes ventricular arrhythmia,” respectively (6, 13–15). Unlike typical CPVT1-associated RyR2 GOF variants that result in a reduced threshold for RyR2 channel activation (i.e., hyperactive channels), these specific RyR2 LOF missense variants lead to reduced channel activity. Notably, despite experiencing exercise-induced cardiac arrest or syncope, the probands hosting 1 of these 3 RyR2 LOF missense variants did not display the hallmark electrical phenotype of CPVT during exercise stress testing, but rather patients’ stress testing was considered normal or with only mild arrhythmias.

Indeed, rather than a GOF in calcium release through hyperactive or hyperreactive (i.e., “leaky”) RyR2 calcium release channels as typically observed in CPVT1, our patient-specific homozygous RYR2 duplication iPSC-CMs displayed a cellular phenotype of CRC deficiency, as evident by severely reduced RyR2 protein levels, essentially no Ca^2+^ responsiveness to ISO, and dramatically reduced Ca^2+^ responsiveness to caffeine (RyR2 agonist).

We have demonstrated that the large homozygous biallelic tandem duplication identified in our pedigrees leads to a significant reduction in *RYR2* transcript and RyR2 protein levels. In fact, we observed a 70% to 80% reduction in *RYR2* mRNA transcript and RyR2 protein expression levels in both homozygous RYR2 duplication iPSC-CMs compared with control iPSC-CMs. The substantial reduction in RyR2 protein expression was confirmed in an autopsy sample of heart tissue from a 12-year-old homozygous RYR2 duplication–positive female relative who died suddenly while playing.

Genomic imbalances referred to as CNVs are defined as deletions or duplications of DNA sequences larger than 1000 bp and can be as large as several megabases in size and be present in a variable copy number compared with the reference genome (16). CNVs involving single or multiple exons of the major channelopathy genes, *KCNQ1*, *KCNH2*, *SCN5A*, and *RYR2*, uncommonly serve as the pathogenic basis of dominantly inherited arrhythmia syndromes such as long QT syndrome, Brugada syndrome, and CPVT (3, 8, 9, 11, 17–19). Smaller genomic imbalances involving single to multiple coding region exons typically result in novel gene transcripts that result in in-frame insertion/deletion mutations that could result in either an LOF or GOF phenotype or frameshift mutations that often lead to early termination and haploinsufficiency through nonsense-mediated decay. However, CNVs may also affect gene expression levels by altering the actual dosage of a particular gene or by indirectly affecting gene transcriptional regulatory elements by positional effect or disruption/duplication of gene repressors, enhancer elements, or gene promoter regions (16, 20).

Whether the large tandem duplication of 344,085 bp involving approximately 26,000 bp of intergenic sequence, *RYR2*’s 5′ UTR/promoter region, and exons 1–4 of *RYR2* identified here results ultimately in a novel fusion protein of RyR2 that contains a tandem repeat of the first 128 amino acids encoded by exons 1–4 of *RYR2* or some other variation thereof, or if this duplication that includes the entire promoter region and 26,000 bp of upstream intragenic sequence simply results in reduced expression of normal RyR2 protein by interfering with a critical *RYR2* transcriptional regulatory element remains to be determined. Our current data illustrate that when present in a homozygous state, the *RYR2* duplication, identified in our patients, results in a greater than 60% reduction in *RYR2* mRNA and RyR2 protein expression.

Interestingly, Yang and colleagues reported in mice that Ryr2-associated cis-element RNA (RACER), a T-box transcription factor–dependent long noncoding RNA located 25,000 bp upstream of *RYR2*, was required for *RYR2* gene expression (21). It is not currently known whether RACER is present in a similar location relative to the human *RYR2* gene and has a similar role in humans. Our patient-specific human iPSC-CM (hiPSC-CM) lines may allow us to further investigate the regulatory role of this duplicated region in the control of *RYR2* gene expression.

While we did not notice a difference in ISO-induced Ca^2+^ sparks or ectopic beating in control iPSC-CMs and RYR2 duplication iPSC-CMs (data not shown), we did observe a substantially reduced Ca^2+^ responsiveness to β-adrenergic stimulation by ISO. In control iPSC-CMs, we saw the expected increase in Ca^2+^ transient amplitude. The ISO-mediated Ca^2+^ peak time and decay time response was indistinguishable between control and patient iPSC-CMs. However, the amplitude response was reduced dramatically (no response to ISO) in patient iPSC-CMs in both independent patient lines compared with control cells. Yet, the peak upstroke time and Ca^2+^ decay time, which translated as activation of RyR2 by PKA and SERCA2a, was not different between control and patient iPSC-CMs. This indicates that the RYR2 channels are functional in the homozygous RYR2 duplication patient lines but consistent with lower RyR2 protein levels than in control lines.

Our control iPSC-CMs displayed the expected abrupt and dramatic increase in intracellular calcium following caffeine treatment. However, neither RYR2 duplication hiPSC-CM line had any significant change in calcium transient amplitude following caffeine. Although RyR2-null mice are embryonically lethal, rodent RyR2-knockout embryonic stem cell–based (22) and RyR2-null mice embryonic cardiomyocyte-based (23) RyR2 functional studies have demonstrated dramatically reduced Ca^2+^ responsiveness to caffeine similar to what has been observed in our RYR2 duplication iPSC-CM lines. However, Bround and colleagues, using a heart-specific inducible RYR2-haploinsufficient (c*Ryr2*Δ50) mouse model with only a single null allele, saw no difference in caffeine-releasable Ca^2+^ between the c*Ryr2*Δ50 and control CMs (24). Importantly, unlike our human patients who are homozygous for the *RYR2* duplication, these heart-specific haploinsufficient mice did not display lethal arrhythmias. This would suggest that the “calcium release deficiency” mechanism responsible for the arrhythmic events in patients homozygous for this duplication may require more than a simple 50% reduction (i.e., haploinsufficiency) in RyR2 protein. In fact, both the patient iPSC-CMs and the heart tissue from one of the sudden death victims revealed a 70%–80% reduction in RyR2 protein.

Interestingly, the reduction in Ca^2+^ responsiveness to caffeine observed in our homozygous RYR2 duplication iPSC-CMs appears different from previous HEK293 cell–based analysis of the aforementioned LOF-associated missense variants, where both p.A4860G and p.S4938F exhibited a normal caffeine response (14, 15). The LOF-associated p.I4855M variant had markedly inhibited caffeine activation, as observed by a dramatic rightward shift in caffeine response (13). This significant rightward shift in caffeine response was also observed with p.I4855M and WT-RYR2 coexpression, suggesting a dominant negative effect of p.I4855M on WT-RYR2 channels (13). Importantly, none of these LOF-associated missense variants has been characterized in a human iPSC-CM model.

Recently, the p.A4860G RyR2 mutation, which was demonstrated to display a dramatic depression in channel activity when expressed in HEK293 cells (25), mainly as a result of loss of luminal Ca^2+^ sensitivity, has been further characterized using a transgenic knockin mouse model (12). Zhao and colleagues demonstrated that ISO-stimulated p.A4860G murine ventricular CMs displayed a decrease in peak of Ca^2+^ release during systole, which gradually overloaded the SR with Ca^2+^. The calcium overload subsequently resulted in random bursts of prolonged Ca^2+^ release, which activated electrogenic Na^+^/Ca^2+^ exchanger activity and triggered EAD (12).

Given the multiple exertion-triggered cardiac events in both patients and several family members who died suddenly during exercise, we conducted FP measurements of the patient RYR2 duplication lines and controls at baseline and following ISO treatment. While there was only a trend toward increased arrhythmic activity in the RYR2 duplication patients’ iPSC-CM lines even without ISO during baseline recording, there was a pronounced and significantly increased arrhythmic behavior in both RYR2 duplication iPSC-CMs versus the control group following ISO treatment (Figure 6), which further worsened the defect and increased the percentage of erratically behaving cells. Additionally, 2 siRNAs’ scramble RNA control were tested, which confirmed that *RYR2* reduction was responsible for the observed anomaly in electrical activity. However, the arrhythmic activity observed with siRNA knockdown of *RYR2* was not as severe as observed in the homozygous *RYR2* duplication iPSC-CMs. This may be explained by resistance of the *RYR2* mRNA transcript to be knocked down below 40% of the control using the 2 siRNAs (the *RYR2* transcript was reduced to ~30% of control in our homozygous patient-derived *RYR2* Dup iPSC-CMs) or length of suppression of siRNA during experimental measurement (72 hours or 1 week after siRNA treatment). This may suggest a certain threshold of *RYR2* transcript/RyR2 protein loss that is required before observing a pathogenic phenotype. The behavior of erratic activity observed in the RYR2 duplication iPSC-CMs seemed to mimic that of DAD activity, which was elicited after full FP generation. Currently, triggered DADs are considered the most accepted cellular mechanism to explain arrhythmias in CPVT (12).

Our single-cell patch-clamp electrophysiology data demonstrated the presence of DADs in both RYR2 duplication hiPSC-CM lines, supporting that this indeed is the cellular mechanism for arrhythmias. Interestingly, one of the RYR2 duplication hiPSC-CM lines also demonstrated a significantly prolonged APD90 (*P* < 0.05). While not consistently observed within the 2 Amish pedigrees, some individuals homozygous for the *RYR2* duplication have been noted to display intermittently prolonged QT intervals (5). We speculate that reduced calcium release from the SR could reduce the calcium-dependent inactivation of the LTCC, thus prolonging phase 2 of the cardiac action potential, resulting in an increase in APD, which may manifest as QT prolongation on the ECG.

Beta blockers have been established as the first line of pharmacological defense in the treatment of CPVT by inhibiting β-adrenergic activity (i.e., exertion, extreme emotion) (26). For patients who continue to break through with cardiac events or experience significant ventricular ectopy on stress testing while on beta blocker therapy, the addition of the antiarrhythmic drug flecainide has been demonstrated to reduce ectopic burden (27). As a proof-of-principle pharmacological therapeutic approach, we treated the patient iPSC-CMs exposed to ISO with 2 commonly prescribed beta blockers, nadolol and propranolol, as well as with flecainide. Both nadolol and propranolol were able to rescue the ISO-treated patient iPSC-CMs by significantly reducing the arrhythmic activity of the cells even below the baseline arrhythmic level of the control iPSC-CMs. Interestingly, treatment with flecainide significantly reduced the arrhythmic activity in the ISO-insulted patient iPSC-CMs; however, the arrhythmic activity remained greater than what was observed in the control cells (Figure 6). This suggests only a partial rescue of the arrhythmic phenotype by flecainide alone was achieved. Given that there is no reliable cardiologic test that can identify an Amish community member as having this homozygous *RYR2* duplication, genotyping followed by prophylactic nadolol/propranolol beta blocker therapy for those positive for the homozygous *RYR2* duplication probably represents the minimal therapy for individuals with this form of CRC deficiency syndrome.

Here, we characterized what we believe is the first iPSC-CM model of a homozygous multiexon *RYR2* duplication mutation identified in 2 large Amish pedigrees associated with exertion-related SUDY/SCA. Unlike the typical GOF mechanism observed in *RYR2-*mediated CPVT, the homozygous multiexon duplication precipitates a “calcium release channel deficiency syndrome” secondary to a profound attenuation in *RYR2* transcription and RyR2 translation, which renders the CICR apparatus nearly insensitive to both catecholamine stimulation and caffeine.

## Methods

### Case description and patient-specific iPSC generation.

Human iPSCs were generated from PBMCs derived from 2 unrelated patients (15-year-old girl and 10-year-old boy) who either survived an exertion-associated SCA and/or have an extensive family history of multiple SUDYs. iPSCs were also generated for 2 unrelated healthy female controls who were negative for the *RYR2* duplication. Figure 1A shows the 2 large multigenerational Amish pedigrees, indicating the family members who provided samples for use in this study. See online supplemental materials for expanded Supplemental Methods sections on reprogramming from PBMCs, quality control assessment of iPSC lines, CM differentiation, CM dissociation, and immunocytochemistry of iPSC-CM germ layers.

### siRNA knockdown of RYR2 studies.

Two human *RYR2* siRNAs were purchased from OriGene Technologies, Inc. (catalog SR304208, GGACUAUAACAGGGCAAAGUGG, CCGAUACAACGAAGUCAUGCAA) and 1 scramble RNA (catalog SR30004). The siRNAs were activated by adding RNase-free duplex buffer based on the manufacturer’s recommendation (OriGene Technologies, Inc.). Using Lipofectamine RNAiMAX (Thermo Fisher Scientific catalog 13778100), 20 μM final siRNAs were transfected into control WT1 iPSC-CMs and incubated for 72 hours based on the manufacturer’s recommendation.

### Real-time quantitative PCR.

Following physical enrichment of iPSC-CMs from beating clusters, total RNA was isolated from multiple biological replicates from all 3 homozygous RYR2 duplication mutant human iPSC-CM clones and control iPSC-CMs. Gene expression levels for *RYR2* and an endogenous control (*GAPDH*) gene were assessed using the 7900 RT-qPCR system (Applied Biosystems, Thermo Fisher Scientific). Relative gene expression analysis was performed using a modified ΔΔCt method employing the Pfaffl formula, which accounts for PCR amplification efficiencies between primer sets. Statistical analysis was performed using GraphPad Prism (GraphPad Software).

### Western blot for relevant cardiac proteins.

Western blot analysis was performed on protein lysates isolated from WT and mutant iPSC-CMs following the detailed methodology outlined in the Supplemental Methods. Proteins were loaded on a 4%–15% TGX gel (Bio-Rad, 456-1083) and transferred onto a PVDF membrane. Membrane was incubated with primary antibody (β-Actin, Santa Cruz Biotechnology, sc-47778; and cardiac RyR2, Thermo Fisher Scientific, MA3-916) diluted at 1:1000 in 5% molecular grade skim milk (Santa Cruz Biotechnology, sc-2325) with TBS with 0.1% Tween-20 (TBS, Bio-Rad, 1706435; and Tween-20, Bio-Rad, 1706531) overnight at 4°C. Finally, the membrane was incubated with SuperSignal West Pico PLUS chemiluminescent ECL substrate (Thermo Fisher Scientific, 34577) for 5 minutes and then exposed to HyBlot CL autoradiography film (Denville Scientific Inc., E3012). Film was scanned and saved as a JPEG file; the relative density of RyR2 to that of β-actin was analyzed and quantified with ImageJ (NIH). There was no significant difference in the protein expression among the lines (data not shown).

### Immunocytochemical analysis of iPSC-CMs.

To demonstrate that the CMs expressed the typical cardiac markers, IF staining for cTnT, cardiac RyR2, and DAPI was performed using the detailed methods outlined in the supplemental materials.

### Quantitative IF in human heart tissue.

Frozen sections of human heart were processed by the Mayo Histology Core lab. Each frozen section was fixed with a cold (–20°C) acetone (MilliporeSigma, 179124) and methanol (Thermo Fisher Scientific, A454-4) (50:50) mixture for 5 minutes at –20°C. The sections were washed immediately at room temperature with PBS to rehydrate tissue. Blocking and primary/secondary antibody staining followed as described above and in the supplemental materials. Imaged data were analyzed by ZEN lite software (ZEISS) to quantify the average intensity that was collected and were analyzed by the statistics software GraphPad Prism (GraphPad Software).

### Fluo-4–measured Ca^2+^ imaging to assess calcium handling.

CM SR Ca^2+^ load and RyR2-mediated leakage were assayed using Fluo-4 fluorescence. Patient and unrelated control iPSC-CMs were dissociated from 24-well plates and then plated and cultured on Geltrex-coated (Gibco, Thermo Fisher Scientific, A1413202), 35 mm, glass-bottom dishes (MatTek, P35G-1.5-10-C) at 37°C, 5% CO_2_, for 3–5 days. CMs were incubated for 30 minutes in DMEM/2% FBS media containing 2 μM of Fluo-4 AM (Thermo Fisher Scientific, F14201) and 0.02% F-127 (Thermo Fisher Scientific, P3000MP). The cells were washed once with fresh DMEM/2% FBS media. During imaging, the dishes were kept in a heated 37°C stage-top environment chamber supplied with 5% CO_2_. Imaging of Ca^2+^ transients was taken under ×40 objective using Nikon Eclipse Ti light microscope. Ca^2+^ transients from single beating iPSC-CMs, paced at 0.25 Hz, were recorded at a speed of 20 ms per frame for 20 seconds at 15% LED power. After finishing baseline recording, appropriate amounts of ISO (MilliporeSigma, I2760) and caffeine (MilliporeSigma, C0750) were added into the recording dish dropwise. The raw data were exported to Excel software (Microsoft) and then analyzed with an in-lab–developed Excel-based program.

### Electrophysiological patch-clamp action potential measurements.

CM aggregate cultures were maintained in DMEM. At differentiation days of 30–60, the iPSC-CMs were subjected to enzymatic dissociation to obtain single-cell suspensions of CMs. These cells were added to 0.1% gelatin-coated glass coverslips maintained in DMEM and stored in a 5% CO_2_ incubator at 37°C before use.

Action potentials from control and RYR2 Dup 1 and RYR2 Dup 2 mutant iPSC-CMs before and after ISO were recorded at room temperature (22°C–24°C) using current clamp mode at a constant rate of 1 Hz through 5 ms depolarizing current injections of 150–500 pA or gap-free mode with the use of an Axopatch 200B amplifier, Digidata 1440A, and pClamp version 10.2 software (Axon Instruments). The extracellular (bath) solution contained (mmol/L) 140 NaCl, 4 KCl, 2 CaCl_2_, 1 MgCl_2_, and 10 HEPES, pH adjusted to 7.4 with NaOH. The pipette solution contained (mmol/L): 110 KCl, 31 KOH, 10 EDTA, 5.17 CaCl_2_, 1.42 MgCl_2_, 4 MgATP, and 10 HEPES, pH adjusted to 7.2 with KOH. Microelectrodes were pulled on a P-97 puller (Sutter Instruments) and fire polished to a final resistance of 2–3 MΩ. Series resistance was compensated by 80%–85%. Data were analyzed using Clampfit (Axon Instruments) and Excel and plotted with Origin 9.1 (OriginLab Corporation).

ISO hydrochloride was purchased from MilliporeSigma, was prepared as 100 μM stock solution in H_2_O, and was diluted into aqueous bath solutions to achieve final concentrations of 100 nM or 1 μM.

### Assessment of FP and arrhythmic activity.

Arrhythmic activity was assessed using an xCELLigence RTCA CardioECR instrument (ACEA Biosciences) at baseline (DMEM/2% FBS media) and during ISO challenge (100 nM). Patient and unrelated control iPSC-CMs were dissociated from 24-well plates and then plated and cultured on fibronectin-coated (Gibco, Thermo Fisher Scientific, 33016-15), 48-well, electronic microtiter plates (ACEA Biosciences, 300600940) at 37°C, 5% CO_2_. FPs were measured 2 times for 20 seconds (block duration) every 10 minutes as a routine baseline measurement.

### Drug rescue of arrhythmic activity.

Various pharmacotherapies were used to test their efficacy on the treatment of arrhythmic events occurring in the mutant iPSC-CMs as a proof-of-principle, treatment-in-a-dish pilot study. Propranolol (1 μM), nadolol (10 μM), or flecainide (6 μM) was administered in combination with ISO (100 nM) immediately before recording. For the drug treatment test, a 1-hour setting was used with a 5-minute interval and 20-second block duration. The percentages of wells presenting arrhythmic activity were recorded per treatment. ISO, propranolol, nadolol, and flecainide were purchased through MilliporeSigma.

### Statistics.

Data points are shown as the mean value, and bars represent the standard deviation unless specially mentioned. A Student’s 2-tailed *t* test was performed to determine statistical significance between 2 groups, and a 1-way ANOVA was performed for comparisons among 3 groups. For multiple post hoc ANOVA analyses, both Tukey’s and Bonferroni’s tests were used. *P* < 0.05 was considered significant.

### Study approval.

Human iPSCs were generated from 2 unrelated patients (15-year-old girl and 10-year-old boy) following receipt of written informed consent for this Mayo Clinic IRB–approved study (09-006465). The heart tissue sample was used following written informed consent from the deceased family member’s parent for this Mayo Clinic IRB–approved study (1216-97).

## Author contributions

DJT contributed to conceptualization of the project, designing of research studies and experiments, data analysis, and writing of the manuscript. CSJK contributed to conceptualization of the project; performed most of the experiments; and contributed to experimental design, data acquirement and analysis, and writing of the manuscript. SKH, DY, and BJO contributed to data acquirement and analysis and writing of the manuscript. HMB, KKF, CMHT, DLA, LAON, IL, and JT contributed to patient sample recruitment, clinical information gathering, and critical review of the manuscript. MJA contributed to conceptualization of the project, designing of research studies and experiments, and writing of the manuscript. All authors discussed the results and commented on the manuscript.

## Supplementary Material

Supplemental data

## Figures and Tables

**Table 1:**
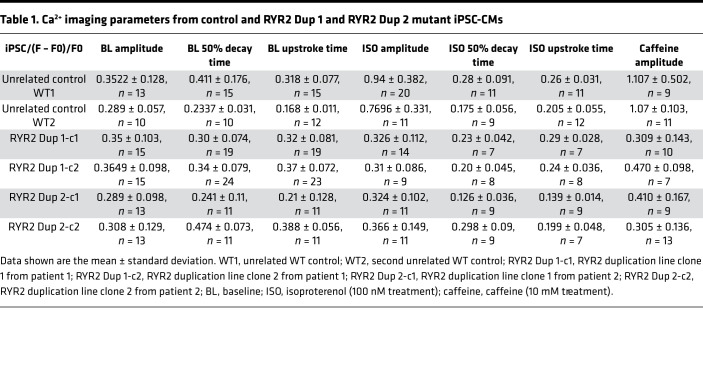
Ca^2+^ imaging parameters from control and RYR2 Dup 1 and RYR2 Dup 2 mutant iPSC-CMs

## Version 1 07/14/2020

In-Press Preview

## Version 2 08/06/2020

Electronic publication
