# Supplementary material for: Molecular characterization of the calcium release channel deficiency syndrome
Source: JCI Insight. 2020 Aug 6;5(15):e135952. doi: 10.1172/jci.insight.135952 (PMC7455073; doi:10.1172/jci.insight.135952)
Supplement: Supplemental data [file jciinsight-5-135952-s078.pdf]

SUPPLEMENTAL MATERIAL

Molecular Characterization of the Calcium Release Channel Deficiency Syndrome

David J. Tester, BS[1*], CS John Kim, PhD[1*], Samantha K. Hamrick, BS[1], Dan Ye, MD[1], Bailey J. O'Hare, BA[1], Hannah M. Bombei, MS, LGC[2], Kristi K. Fitzgerald, MS[3], Carla M. Haglund-Turnquist[1], Dianne Atkins, MD[2], Luis A. Ochoa Nunez, MD[2], Ian Law, MD[2], Joel Temple, MD[3], Michael J. Ackerman, MD, PhD[1]

***Co-equal first authors**

[1]Department of Molecular Pharmacology & Experimental Therapeutics/Windland Smith Rice Sudden Death Genomics Laboratory; Department of Cardiovascular Medicine/Division of Heart Rhythm Services; Department of Pediatric and Adolescent Medicine/Division of Pediatric Cardiology, Mayo Clinic, Rochester, Minnesota.

[2]Division of Pediatric Cardiology, University of Iowa, Stead Family Children's Hospital, Iowa City, Iowa.

[3]Nemours Cardiac Center/Alfred I. duPont Hospital for Children, Wilmington, Delaware.

Correspondence:

Michael J. Ackerman, M.D., Ph.D.

Mayo Clinic Windland Smith Rice Sudden Death Genomics Laboratory

Guggenheim 501, Mayo Clinic, Rochester, MN 55905

507-284-0101 (phone), 507-284-3757 (fax), ackerman.michael@mayo.edu, @MJAckermanMDPhD

SUPPLEMENTAL METHODS

Reprogramming from Peripheral Blood Mononuclear Cells (PBMCs)

PBMCs were processed by the Mayo Clinic's Regenerative Medicine Biotrust facility and stored in liquid nitrogen until further use at approximately $2x10^6$ PBMCs per vial. A 37°C water bath was used for quick thawing of frozen PBMCs. The PBMCs were transferred immediately into cold base DMEM (Corning, 10-013-CV) or RPMI (Gibco, 11875-093) media and centrifuged at 800g for 5min to dilute out freezing solution. Blood media (IMDM base PBMC activating media) was used to grow PBMCs for 3 to 7 days in a 5% CO2, 37°C, humidified incubator. Briefly, Blood media is composed with IMDM (Gibco, 12440053), 20% KnockOut Serum Replacement (Gibco, 10828010), IGF-1 (Peprotech, 100-11, 100µg/mL), EPO (Peprotech, 100-64, 10µg/mL), IL-3 (Peprotech, 200-03, 10µg/mL), and hSCF (Peprotech, 300-07, 50µg/mL). A CytoTune 2.0 Kit (Invitrogen, A16517), which includes MOI 5:5:3 (KOS:c-Myc:Klf4), was used for $1x10^5$ PBMCs with a 90min, 1500g, 25°C centrifugal transduction. Media was changed with 50:50 of Blood media and ES media 48h after transduction. Briefly, ES media is composed of KnockOut DMEM (Gibco, 10829018) with 20% KnockOut Serum Replacement (Gibco, 10828010), without antibiotics. Four days after transduction, media was replenished by ES media only. Seven days after transduction, media was replenished with mTeSR1 only. mTeSR1 media was changed every other day until seeing visible iPSC colonies. Colonies were picked within 21 days post infection with Yamanaka factors. Two representative clones were further expanded and characterized for each patient-specific mutant line.

Quality Control Assessment of iPSC Lines

Karyotyping

Karyotyping for each of the patient's iPSC clones was completed by Mayo Clinic's Cytogenetics Laboratory according to Mayo Clinic IRB (09-006465). All mutant iPSC clones tested demonstrated normal karyotypes (**Supplemental Figure 1**).

Testing for Mycoplasma Contamination

The iPSCs were tested for the presence of mycoplasma contamination using the Universal Mycoplasma Detection Kit (ATCC® 30-1012K™).

RYR2 Duplication Variant Confirmation in iPSC Lines

Genomic DNA was isolated from each iPSC clone using the DNeasy Blood & Tissue kit (Qiagen, 69504). Sanger sequencing using forward (CTAGCCCTTTTCTAACCTATACCATTGG) and reverse (CATTGAAAGCTACATACCATTCTGGAG) PCR primers specific to the unique junction formed at the site of the tandem duplication were used to confirm the duplication variant. Additionally, *RYR2* exon 1-4 duplication genotyping of all iPSC clones was performed using the commercially available TaqMan® Copy Number Assay, Hs00134396_cn (ThermoFisher Scientific, 4400291) which maps to *RYR2* exon 2 and the TaqMan® Copy Number Reference Assay, human, RNAse P (ThermoFisher Scientific, 4403326) according to the manufacturer's instructions using an Applied Biosystems ViiA 7 Real-Time PCR System with ViiA7 Software v1.2.

Briefly, each sample underwent real-time PCR amplification using four replicates per sample using TaqMan® Genotyping Master Mix (ThermoFisher Scientific, 4371353). Three

reference samples with known duplication status (i.e. homozygous wild-type, heterozygous *RYR2* duplication, and homozygous *RYR2* duplication) were included in each assay run. Following amplification, the data was first analyzed using QuantStudio^{TM} Real-Time PCR system Software v1.3 (Applied Biosystems) set to a manual CT threshold of 0.2 and auto baseline set to on and then transferred to CopyCaller^{TM} software (Applied Biosystems) for automated CNV calling.

Immunostaining of iPSCs for Pluripotent Markers

All iPSC clones, including the unrelated wild-type control lines, were confirmed to express TRA-1-81 (R&D Systems, MAB8495), Nanog (ThermoFisher, PA1-097X), and SSEA-4 (ThermoFisher, MA1-021) pluripotent markers (**Supplemental Figure 1**). The iPSCs were cultured on a 24-well glass bottom plate (Cellvis, P24-1.5H-N) and fixed with ice cold 4% paraformaldehyde (ThermoScientific, J19943-K2) in PBS (pH 7.0) for 10 minutes at room temperature (RT), washed 3 times with PBS and then permeabilized with 0.1% Triton X-100 (Sigma, T9284)/PBS (Gibco, 14190-144) (0.1% PBST) and 5% goat serum (Gibco, 16210-064) for 30 minutes at RT. The cells were stained with primary antibodies at a concentration of 1µg/mL in 0.1% PBST and 5% goat serum for 1 hour at RT. The cells were then washed in 0.1% PBST and incubated for 15 minutes, 3 times. The cells were then stained with Alexa Fluor 488 goat anti-rabbit (Invitrogen, A11008) or 594 goat anti-mouse (Invitrogen, A11005) secondary antibodies at a dilution of 1:200 and DAPI (Invitrogen, D1306) was added to each secondary antibody solution at a concentration of 1µg/mL. The cells were then washed in 0.1% PBST and incubated for 15 minutes, 3 times. The cells were maintained in PBS and stored in the dark at 4°C until imaging. Images were acquired on a Zeiss LSM 780 confocal microscope in the Mayo Clinic Microscopy and Flow Cytometry Cell Analysis Core Facility.

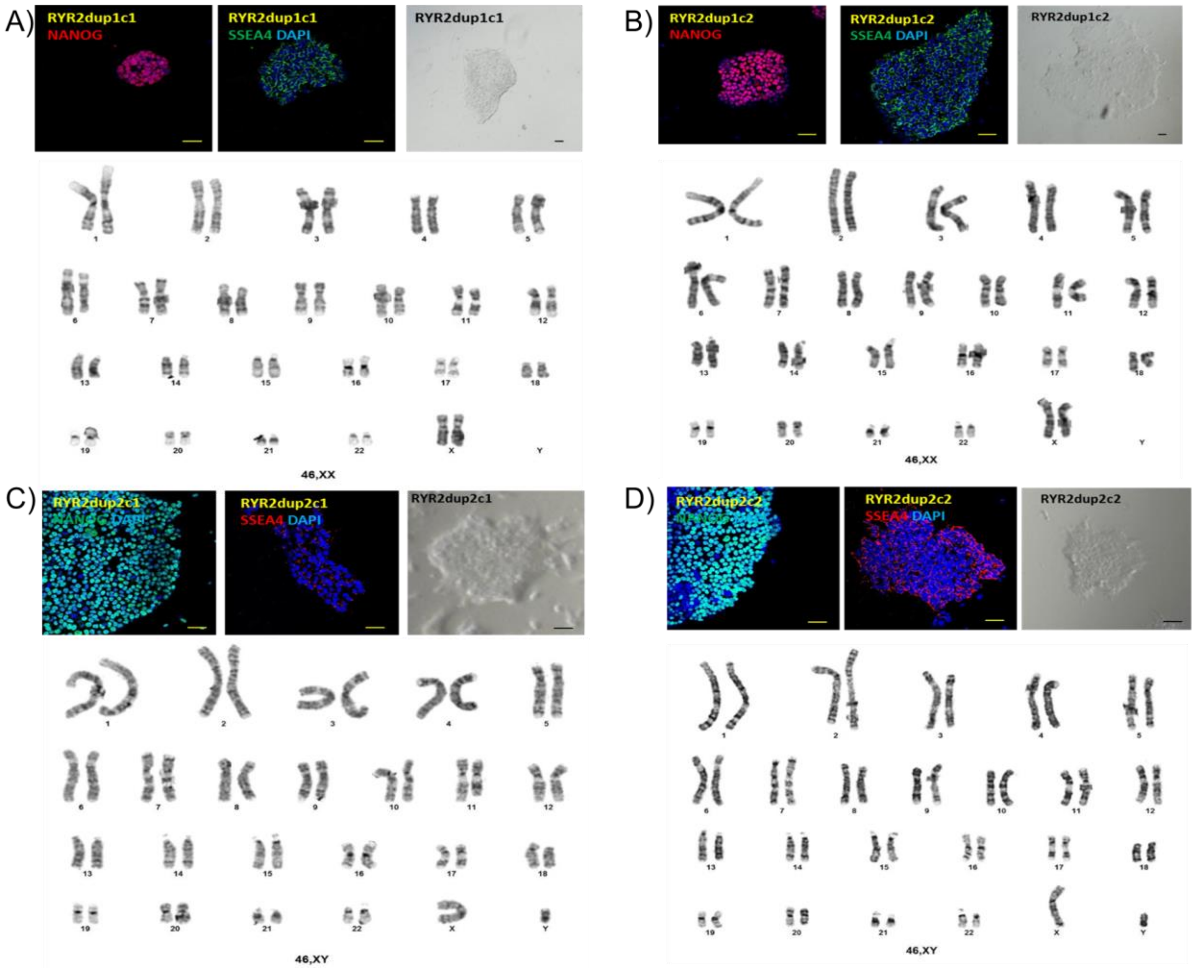

Supplemental Figure 1. **Generation and confirmation of both patient-specific RYR2 duplication iPSCs**. A. RYR2dup1 clone1, B. RYR2dup1 clone2, C. RYR2dup2 clone1, D. RYR2dup2 clone2. Upper panel, pluripotent markers and phase-contrast light images of iPSC colonies from each clone of the patient lines used for the study. Scale bars equal 50µm. Bottom panel, normal karyotype of different patient hiPSCs.

Cardiomyocyte Differentiation

The iPSCs were cultured in mTeSR™ (STEMCELL Technologies, 85851) in 6-well plates (Falcon, 353046) pre-coated with Geltrex (Life Technology, A1413302) and incubated at 37°C and 5% CO_2. The mTeSR media was changed daily. At 85% confluence, iPSCs were disaggregated with TrypLE™ Express (Life Technologies, 12605-010), seeded into 24-well plates (Corning, 3524), cultured in mTeSR™ plus 10µM ROCK inhibitor (Tocris, 1254) and allowed to grow for 2-4 days until 80-90% confluent. The differentiation strategy used has been reported previously (Burridge & Wu, 2014). For differentiation, the culture medium was changed to RPMI 1 640 GlutaMAX™ plus 25mM HEPES (Life Technologies, 72400120) supplemented with B27-minus insulin (Gibco, A18956-01) containing 5µM CHIR99021 (Sigma, SML1046) for 48 hours (Day 2). After 48 hours, the medium was changed to RPMI-B27-minus insulin containing 5 µM IWP2 (Tocris, 3533) and incubated until day 4. On day 4, the medium was changed back to RPMI 1640 GlutaMAX™ plus 25mM HEPES supplemented with B27-minus insulin and cells were maintained in this media until beating cardiomyocytes (iPSC-CMs) appeared, typically around day 10 or day 12. One week following the initial observation of beating, the media was changed and the iPSC-CMs were maintained in DMEM with 4.5g/L, L-glutamine, and sodium pyruvate (Corning, 10-0013-CV), supplemented with 2% FBS (Gibco, 10437-028).

Cardiomyocyte Dissociation

Cardiomyocyte aggregate cultures were maintained in 2% FBS/DMEM media in 24-well plates. At differentiation day 30, iPSC-CMs were subjected to enzymatic dissociation using cardiomyocyte dissociation media (STEMCELL Technologies, 05025) to obtain single cell

suspensions of CMs. The iPSC-CMs were first washed with 1 mL of PBS for 20-30 seconds. Following aspiration of the PBS, 250 µL of cardiomyocyte dissociation media was added to each well. After 2 minutes of incubation, the cardiomyocyte dissociation media was aspirated and the cells were incubated for 4-8 minutes in a 5% CO_2 incubator at 37^oC. Following incubation, 500 µL of DMEM with 20% FBS was added to each well. The solution was titrated 3-5 times with a 1000 µL pipet tip and cells were transferred to a Geltrex-coated 35 mm glass bottom dish (MatTek, P35G-1.5-10-C) and cultured at 37^oC, 5% CO_2 for 24 hours. The media was then changed to DMEM with 2% FBS and stored in a 5% CO_2 incubator at 37^oC until use.

Immunocytochemistry of iPSC-CM Germ Layers

To test for the formation of separate germ layers, cells were harvested from wells containing 10-15 day old differentiated iPSC-CMs using the STEMCELL Technologies protocol previously described and plated into glass bottom 24-well plates. Cells were allowed to grow for 5 days, then subjected to fixation with 4% paraformaldehyde for 10 minutes at room temperature (RT) followed by being washed 3 times with PBS. The iPSC-CMs were then permeabilized/blocked with 0.1% Triton X-100/ PBS (PBST)/5% goat serum for 45 minutes at RT, and subsequently incubated in primary antibody solution made of PBST/5% goat serum containing a 1:250 dilution of neuron (ectoderm)-specific TUJ-1/β-III-tubulin (Biotechne, MAB1195), sarcomere (mesoderm)-specific cTNT (Abcam, ab45932) and/or α-actinin (Sigma, A7811-.5ML), and endoderm-specific SOX17 (ThermoFisher, PA5-23352) primary antibodies at RT overnight. The next day, cells were washed 3 times with PBST/5% goat serum at RT before being incubated in PBST/5% goat serum with a 1:250 dilution of Alexa Fluor® 488 goat-anti-rabbit (Invitrogen, A11008) and Alexa Fluor 594 goat-anti-mouse (Invitrogen, A11005) secondary antibodies at RT for 60 minutes; DAPI was added to each secondary antibody solution at a concentration of

1µg/mL. After secondary antibody incubation, cells were washed 3 times with PBST and finally covered in 500 µL of PBS for imaging. Images were acquired on a Zeiss LSM 780 confocal microscope in the Mayo Clinic Microscopy and Flow Cytometry Cell Analysis Core Facility (**Supplemental Figure 2**). For iPSC-CM cell size analysis, acquired images of single, cardiac marker-positive cells were analyzed using ImageJ Software to assess surface area as an indication of cell size (National Institutes of Health, Bethesda, MD).(1-3)

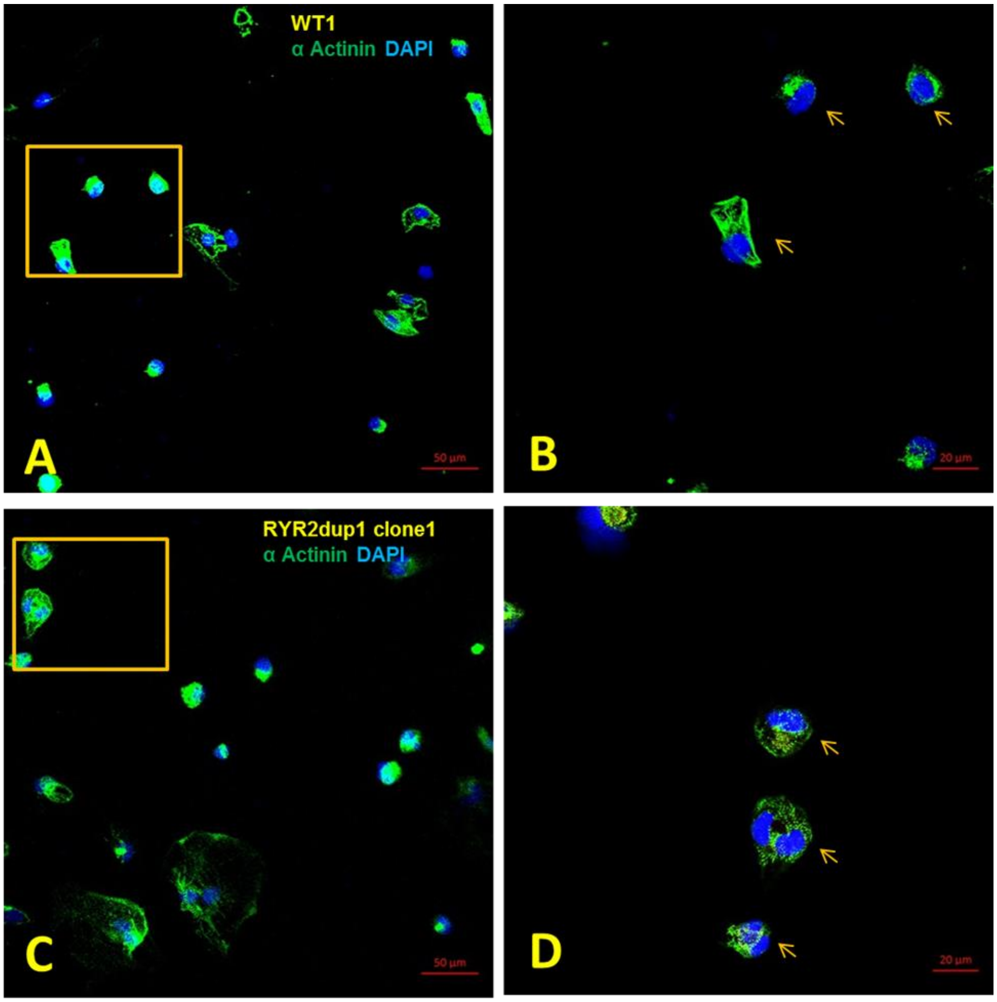

Supplementary Figure 2. Representative image from differentiated iPSC-CMs. Cardiac differentiation and enrichment method showed pure cardiomyocytes (α-actinin positive cell divided by DAPI positive). Pictures are randomly taken in low magnification followed by further

imaging to show striation. A. WT1 staining image B. high magnification image from rectangular region from A. C. RYRdup1 staining image D. high magnification from rectangular region from C.

Real-Time Quantitative Polymerase Chain Reaction

Following physical enrichment of iPSC-CMs from beating clusters, total RNA was isolated from multiple biological replicates from all four homozygous RYR2 duplication mutant hiPSC-CM clones and control iPSC-CMs using the mirVana™ miRNA isolation kit (Invitrogen, AM1560) according to the manufacturer's instruction. The cDNA was prepared from 60 ng of RNA using the QuantiTect Reverse Transcription Kit (Qiagen, 205311) according to the manufacturer's instruction. Gene expression levels for *RYR2* and an endogenous control (*GAPDH*) gene was performed using the 7900 Real-time (RT) polymerase chain reaction (PCR) system (Applied Biosystems). Relative gene expression analysis was performed using a modified ΔΔCt method employing the Pfaffl formula which accounts for PCR amplification efficiencies between primer sets. Statistical analysis was performed using GraphPad Prism (GraphPad Software).

Primer sequences used in RT-qPCR

Name	Sequence (5' to 3')
RYR2-Forward	GGAGTCCACTTCCAATTCCA
RYR2-Reverse	CTCTTGCAAGCCAACATCAA
GAPDH-Forward	GAGTCAACGGATTTGGTCGT
GAPDH-Reverse	GACAAGCTTCCCGTTCTCAG
cTnT-Forward	TTCACCAAAGATCTGCTCCTCGCT
cTnT-Reverse	TTATTACTGGTGTGGAGTGGGTGTGG

Western Blot for Relevant Cardiac Proteins

iPSC-CMs were lysed in RIPA buffer (EMD Millipore Corp., 20188) with Protease Inhibitor Cocktail (TaKaRa, 635673), kept on ice for 30 min, sonicated six times for 3 seconds each time, and then centrifuged at 14000 rpm for 15 minutes at 4°C. Supernatant was removed to a new tube. Prior to gel loading, lysate was mixed with 1:1 with 2X loading buffer (950 µL of 2X Laemmli + 50 µL of 2-Mercaptoethanol; Laemmli sample buffer, Bio-Rad, 1610737 and 2-Mercaptoethanol, Bio-Rad, 1610710), denatured at 95°C for 5 minutes, then immediately placed on ice. Lysates were aliquoted and kept in -80°C freezer. Proteins were loaded on 4-15% TGX gel (Bio-Rad, 456-1083) and transferred onto PVDF membrane using Trans-Blot Turbo RTA Transfer Kit (Bio-Rad, 1704272). Membrane was incubated with primary antibody (Troponin T (cTnT), Abcam, ab45932, alpha-actinin, Sigma, A7811, and cardiac ryanodine receptor-2 (RyR2), ThermoFisher, MA3-916) diluted at 1:1000 in 5% molecular grade skim milk (Santa Cruz, sc-2325) with TBS-T buffer (TBS with 0.1% Tween 20; TBS, Bio-Rad, 1706435 and Tween 20, Bio-Rad, 1706531) overnight at 4°C. The next day, the membrane was washed with TBS-T buffer for 45 minutes prior to being incubated in secondary antibody (goat anti-rabbit, Invitrogen, 65-6120 or goat anti-mouse, R&D Systems, HAF007) diluted 1:2000 in 5% molecular grade skim milk with TBS-T, for 1 hour at RT. The membrane was then washed in TBS-T for 30 minutes. Finally, the membrane was incubated with SuperSignal™ West Pico PLUS chemiluminescent ECL substrate (ThermoFisher, 34577) for 5 minutes and then exposed to HyBlot CL autoradiography film (Denville Scientific Inc., E3012). Film was scanned and saved as JPEG file; the relative density of RyR2 to that of β-Actin was analyzed and quantified

with ImageJ (National Institutes of Health, Bethesda, MD). There was no significant difference in the protein expression among the lines (data not shown).

Immunocytochemical Analysis of iPSC-CMs

To demonstrate that the CMs express the typical cardiac markers, immunofluorescence staining for cardiac troponin (cTnT), cardiac ryanodine receptor-2 (RyR2), and DAPI was performed. Following cardiomyocyte dissociation from 24-well plates, the iPSC-CMs were cultured on a 24-well glass bottom plate and fixed with ice cold 4% paraformaldehyde in PBS (pH 7.0) for 10 minutes at room temperature (RT), washed 3 times with PBS. Permeabilization performed with PBS-T (0.1% Triton X-100/PBS) and 5% goat serum for 60 minutes at RT, then incubated with 1:200 dilution of Troponin T (cTnT, Abcam, ab45932), 1:200 dilution of cardiac ryanodine receptor-2 (RyR2, ThermoFisher, MA3-916), 1:200 dilution of calreticulin (Abcam, ab2907), 1:200 dilution of calsequestrin-2 (Casq2, Invitrogen, PA5-21706), or 1:200 alpha-actinin (Sigma, A7811) overnight at 4°C. The next day, cells were washed 3 times with PBS-T, incubated with 1:500 dilution of Alexa Fluor 488 goat anti-rabbit (ThermoFisher, A11008) and Alexa Fluor 568 goat-anti-mouse (ThermoFisher, A11004) at RT for 60 minutes and washed 3 times with PBS-T. Images were acquired on a Zeiss LSM 780 confocal microscope in the Mayo Clinic Microscopy and Flow Cytometry Cell Analysis Core Facility.

Quantitative Immunofluorescence in Human Heart Tissue

Frozen sections of human heart were processed by the Mayo Clinic Histology Core Laboratory. Frozen section was fixed with a cold (-20 ^{o}C) acetone (Sigma, 179124) and methanol (Fisher, A454-4) (50:50) mixture for 5 minutes at -20° C. The sections were washed immediately at room temperature with PBS to re-hydrate tissue. Blocking and primary/secondary antibody

staining was followed as described above. Imaged data was analyzed by ZEN lite software (Zeiss) to quantify the average intensity that was collected and was analyzed by the statistic software, GraphPad Prism (GraphPad Software).

Fluo-4-Measured Ca^{2+} Imaging to Assess Calcium Handling

Cardiomyocyte sarcoplasmic reticulum (SR) Ca^{2+} load and RyR2-mediated leakage were assayed using fluo-4 fluorescence. Patient and unrelated control iPSC-CMs were dissociated from 24-well plates and then plated and cultured on Geltrex-coated (Gibco, A1413202) 35mm glass bottom dishes (MatTek, P35G-1.5-10-C) at 37°C, 5% CO_2 for 3-5 days. Cardiomyocytes were incubated for 30 minutes in DMEM/2%FBS media containing 2 μM of Fluo-4 AM (ThermoFisher, F14201) and 0.02% F-127 (ThermoFisher, P3000MP). The cells were washed once with fresh DMEM/2%FBS media. During imaging, the dishes were kept in a heated 37°C stage-top environment chamber supplied with 5% CO_2. Imaging of Ca^{2+} transients was taken under 40X objective using Nikon Eclipse Ti light microscope. Ca^{2+} transients from single beating iPSC-CMs, paced at 0.25Hz were recorded at speed of 20ms per frame for 20 seconds at 15% LED power. After finishing baseline recording, an appropriate amount of isoproterenol (ISO) (Sigma, I2760) and caffeine (Sigma, C0750) were added into recording dish dropwise. The raw data was exported to Excel software (Microsoft, Redmond, WA) and then analyzed with an in-lab developed Excel-based program.

Electrophysiological Patch-Clamp Action Potential Measurements

Cardiomyocyte aggregate cultures were maintained in DMEM media. At differentiation days of 30-60, the enriched iPSC-CMs were subjected to enzymatic dissociation to obtain single cell suspensions of cardiomyocytes. These cells were added to 0.1% gelatin coated glass coverslips maintained in DMEM media and stored in a 5% CO_2 incubator at 37°C before use.

Action potentials (AP) from control, RYR2dup1 and RYR2dup2 mutant iPSC-CMs before and after isoproterenol (ISO) were recorded at room temperature (22-24°C) using current clamp mode at a constant rate of 1 Hz through 5 ms depolarizing current injections of 150-500 pA[1, 2] or gap free mode with the use of an Axopatch 200B amplifier, Digidata 1440A and pClamp version 10.2 software (Axon Instruments, Foster City, CA). The extracellular (bath) solution contained (mmol/L): 140 NaCl, 4 KCl, 2 CaCl2, 1 MgCl2, and 10 HEPES, pH adjusted to 7.4 with NaOH. The pipette solution contained (mmol/L): 110 KCl, 31 KOH, 10 EDTA, 5.17 CaCl2, 1.42 MgCl2, 4 MgATP and 10 HEPES, pH adjusted to 7.2 with KOH. Microelectrodes were pulled on a P-97 puller (Sutter Instruments, Novato, CA) and fire polished to a final resistance of 2-3 MΏ. Microelectrodes were pulled on a P-97 puller (Sutter Instruments, Novato, CA) and fire polished to a final resistance of 2-3 MΏ. Series resistance was compensated by 80-85%. Data was analyzed using Clampfit (Axon Instruments), Excel (Microsoft, Redmond, WA), and plotted with Origin 9.1 (OriginLab Corporation, Northampton, MA).

Isoproterenol hydrochloride (ISO) was purchased from Sigma-Aldrich (St. Louis, MO), prepared as 100 µM stock solution in H_2O and was diluted into aqueous bath solutions to achieve final concentrations into 100 nM or 1 µM.

Assessment of Field Potential and Arrhythmic Activity

Arrhythmic activity was assessed using an xCELLigence® RTCA CardioECR instrument (ACEA Biosciences) at baseline (DMEM/2%FBS media) and during ISO challenge (100 nM). Patient and unrelated control iPSC-CMs were dissociated from 24-well plates and then plated and cultured on fibronectin-coated (Gibco, 33016-15) 48-well electronic microtiter plates

(ACEA Biosciences, 300600940) at 37°C, 5% CO_2. Field potentials (FPs) were measured two times for 20 seconds (block duration) every 10 minutes as a routine baseline measurement.

Each 20 seconds recording counted as independent experiment. We counted the erratic electrical activity showing electrodes and divided this by total number of recording electrodes per experiment. For example, if no electrode showed erratic activity then we assigned a value of Zero and if 1 electrode out of 4 showed erratic activity, then we assigned a value of 0.25. Overall, this normalization give how many beatings show erratic activity in given time.

Drug Rescue of Arrhythmic Activity

Various pharmacotherapies were used to test their efficacy on the treatment of arrhythmic events occurring in the mutant iPSC-CMs as a proof-of-principle, treatment-in-a-dish pilot study. Propranolol (1µM), nadolol (10µM) or flecainide (6µM) were administered in combination with ISO (100 nM) immediately before recording. For the drug treatment test, a 1 hour setting was used with a 5 minute interval and 20 second block duration. The percentages of wells presenting arrhythmic activity were recorded per treatment. Isoproterenol, propranolol, nadolol, and flecainide were purchased through Sigma/Millipore.

Statistical Analysis

Data points are shown as the mean value and bars represent the standard deviation unless specifically mentioned. A Student's t-test was performed to determine statistical significance between two groups and a one-way ANOVA was performed for comparisons among three groups. For multiple post-hoc ANOVA analyses, both Tukey's and Bonferroni tests were used. A $p<0.05$ was considered to be significant.

SUPPLEMENTAL RESULTS

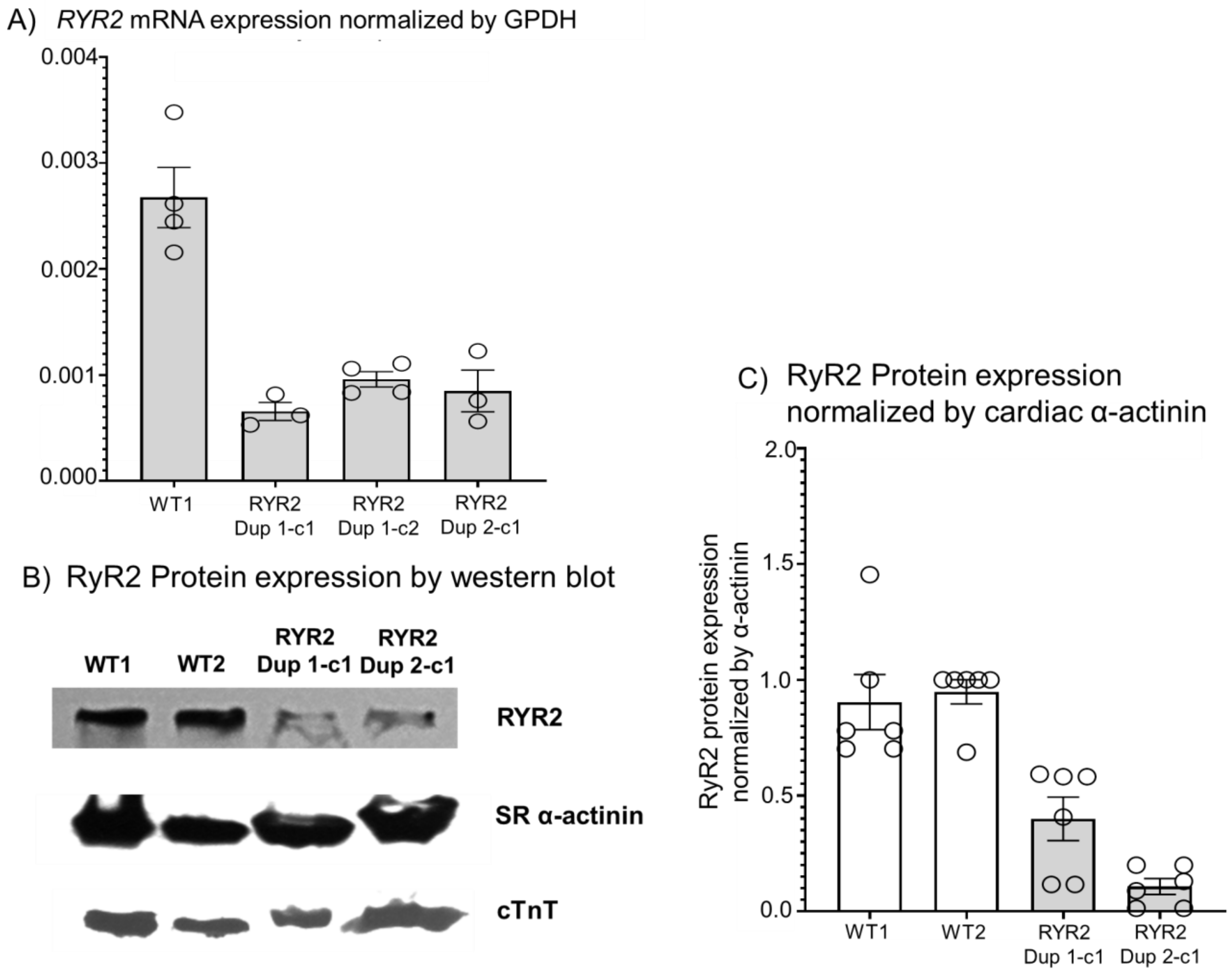

Supplemental Figure 3. Reduced RyR2 protein in patient iPSC-CMs Shown are A) RT-qPCR of *RYR2* mRNA transcript normalized by GAPDH for wild-type (WT) control and homozygous RYR2 duplication iPSC-CMs for both patient 1 (RYR2 Dup 1-clone 1 and -clone 2) and patient 2 (RYR2 Dup 2-clone 1), B) Representative western blots with RyR2 , α-actinin, and cTnT antibodies for two unrelated controls (WT1 and WT2) and for both patient 1 (RYR2 Dup 1-c1) and patient 2 (RYR2 Dup 2-c1) iPSC-CMs and C) a summary graph of RyR2 protein expression normalized to α-actinin. Four independent RT-qPCR experiments were performed per sample. Six independent western blot experiments were performed per sample.

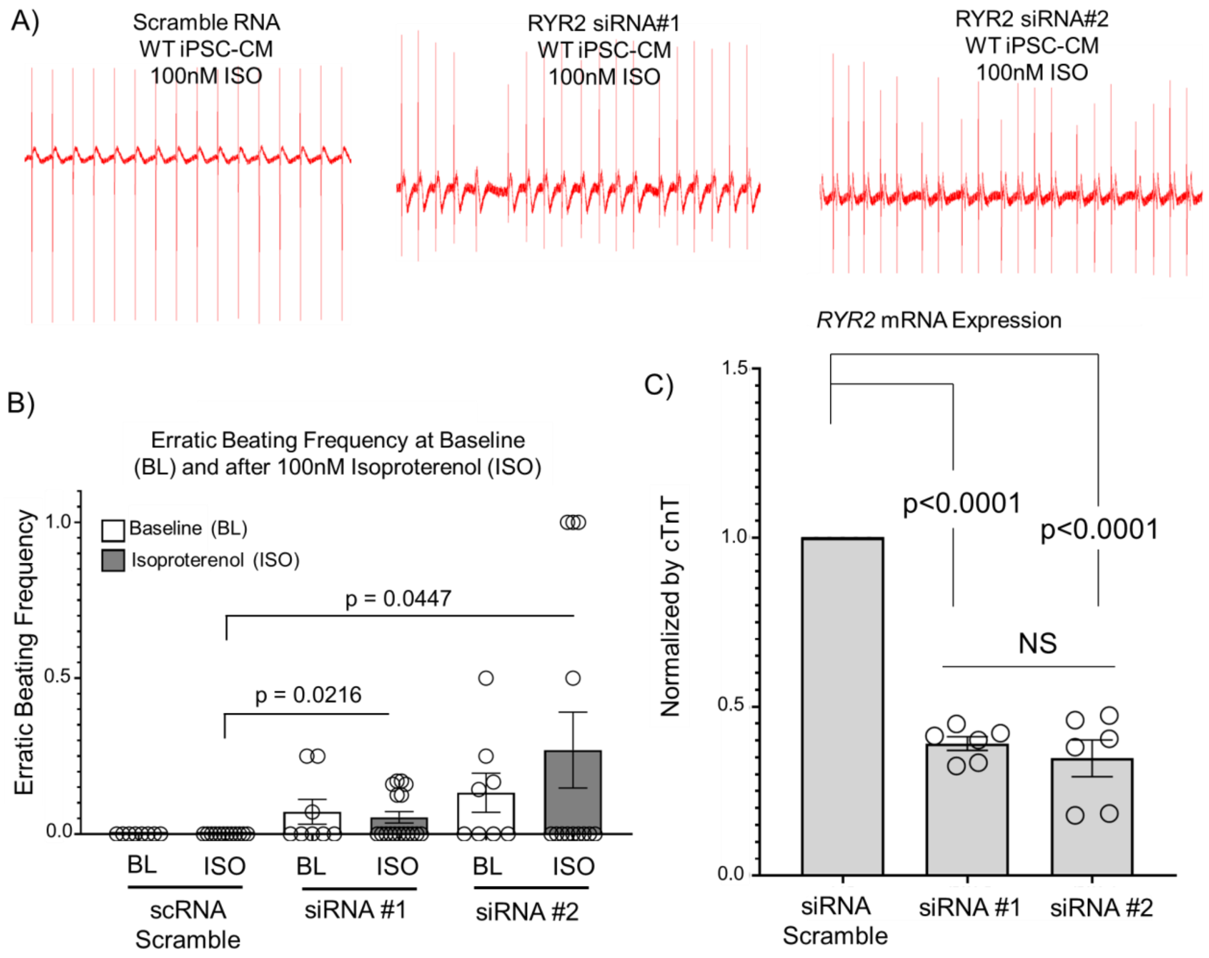

Supplemental Figure 4. Erratic Beating Frequency in siRNA *RYR2* Knockdown iPSC-CMs
Shown in A are representative field potential (FP) recordings from scramble (sc) RNA, siRNA #1, and siRNA #2 treated normal iPSC-CMs following ISO (100nM) treatment. Panel B depicts a bar graph summary showing the erratic beating frequency (i.e. arrhythmic events) present at baseline and following ISO treatment in scRNA, siRNA #1, and siRNA #2 treated iPSC-CMs. The number of responding electrodes for recording for each sample and treatment ranged from n=21 to n=141. Four different date/batches of independent experiments were performed. Shown in panel C is the RT-qPCR of *RYR2* mRNA transcript normalized by cardiac troponin T (cTnT). Three independent technical replicates were included (N=6) for each RT-qPCR experiment. A Student's t-test was performed to determine statistical significance between two groups. A $p<0.05$ was considered to be significant.

Supplemental Table 1. Action potential parameters from control, RYR2-Dup1 and RYR2-Dup2 mutant iPSC-CMs

	MDP (mV)	Amplitude (mV)	APD50 (ms)	APD90 (ms)
Unrelated Control (n=10)	-56.2±2.6	86.9±4.0	305.9±39.7	420.8±45.9
RYR2 Dup1-c2 (n=10)	-52.8±4.4	86.7±6.5	344.0±35.9	428.8±40.0
RYR2 Dup2-c2 (n=6)	-48.3±5.1	80.1±8.1	416.8±80.5	612.0±69.4*

RYR2 Dup1-c2 = RYR2 duplication from patient 1; clone 2; RYR2 Dup2-c2 = RYR2 duplication from patient 2; clone 2; MDP = maximum diastolic potential, APD = action potential duration, * = $p<0.05$

SUPPLEMENTAL REFERENCES

1. Schindelin J, et al. Fiji: an open-source platform for biological-image analysis. *Nat Methods.* 2012;9:676.
2. Kadota S, Pabon L, Reinecke H, and Murry CE. In vivo maturation of human induced pluripotent stem cell-derived cardiomyocytes in neonatal and adult rat hearts. *Stem Cell Reports.* 2017;8(2):278-89.
3. Tanaka A, et al. Endothelin-1 induces myofibrillar disarray and contractile vector variability in hypertrophic cardiomyopathy-induced pluripotent stem cell-derived cardiomyocytes. *J Am Heart Assoc.* 2014;3(6):e001263.
